# Efficacy and safety of traditional Chinese medicine decoctions in breast cancer treatment: a network meta-analysis

**DOI:** 10.3389/fonc.2026.1785268

**Published:** 2026-07-08

**Authors:** Haidi Han, Yanan Du, Juncai Li, Yanjing Dong, Jingyu Zhu, Haixia Wang

**Affiliations:** Shandong Second Medical University, Weifang, China

**Keywords:** breast cancer, decoction, efficacy, network meta-analysis, safety, traditional Chinese medicine

## Abstract

**Background:**

Breast cancer is the most common malignant tumor among women globally. While traditional treatments are effective, they are associated with numerous side effects. In recent years, traditional Chinese herbal decoctions have garnered attention as adjunctive therapeutic approaches. This study employs a network meta-analysis to evaluate the efficacy and safety of various Chinese herbal decoctions in breast cancer treatment.

**Methods:**

A systematic search was conducted across PubMed, Embase, Cochrane Library, Web of Science, CNKI, Wan fang, and VIP databases for randomized controlled trials comparing the efficacy and safety of different Chinese herbal decoctions in breast cancer patients, up to 20 December 2025. Quality assessment was performed using the Risk of Bias 2.0 tool. Network meta-analysis was conducted using R 4.5.1.

**Results:**

A total of 68 articles involving 4,708 patients were included, meta-analysis results suggest that compared with the control group, for objective response rate YHHYT [OR = 4.76, 95%CrI (1.25, 25)] ranked first in cumulative probability (81.42%). Regarding disease control rate, BZT demonstrated an [OR = 5.77, 95%CrI (1.59, 29)] and ranked first in cumulative probability (74.66%). For CD4+/CD8+, FJHQT [MD= MD = 0.69, 95%CrI (0.2, 1.17)] achieved an 88.00% cumulative probability ranking, finally, regarding nausea and vomiting relief BZT [OR = 0.25, 95%CrI (0.1, 0.57)] ranked first in cumulative probability (75.67%).

**Conclusions:**

This study employed a network meta-analysis to evaluate the efficacy of multiple Chinese herbal decoctions in breast cancer treatment. Results demonstrated that these decoctions exhibited significant therapeutic effects in enhancing target response rates, disease control rates, and immune function (CD4+/CD8+ ratio) among breast cancer patients, while also alleviating chemotherapy side effects such as nausea and vomiting.

**Systematic Review Registration:**

https://www.crd.york.ac.uk/PROSPERO/view/CRD420261279914, identifier CRD420261279914.

## Background

Breast cancer ranks among the most prevalent malignant tumors affecting women globally. According to World Health Organization (WHO) statistics, its incidence has risen annually, posing a significant threat to women’s health (1). As per Global Cancer Statistics (GLOBOCAN 2020), annual new cases of breast cancer now exceed two million, accounting for nearly a quarter of all new cancer diagnoses (2, 3). Treatment approaches primarily encompass surgery, radiotherapy, chemotherapy, targeted therapy, and immunotherapy (4). However, despite certain advances in breast cancer management through these conventional methods, numerous issues and challenges persist (5, 6). These include inconsistent treatment outcomes, risks of recurrence and metastasis, treatment-related side effects, and compromised patient quality of life (7). Consequently, exploring and developing more effective, comprehensive, and personalized therapeutic strategies has emerged as a key focus in contemporary breast cancer research and treatment.

Within adjuvant breast cancer therapy, traditional Chinese medicine (TCM) has garnered increasing attention due to its extensive historical heritage and rich clinical experience (8). TCM theory posits that breast cancer development is closely linked to factors such as qi and blood disharmony, yin-yang imbalance, and organ dysfunction within the body. Consequently, regulating the internal environment through herbal medicine to restore the body’s natural equilibrium has emerged as a viable adjunctive therapeutic approach. As a common form of TCM treatment for breast cancer, decoctions exhibit characteristics such as syndrome differentiation and individualized regulation (9, 10). They can mitigate side effects from conventional therapies like chemotherapy and radiotherapy to a certain extent, thereby improving patients’ quality of life and enhancing overall treatment efficacy.

In recent years, an increasing body of clinical research and experimental data has demonstrated the therapeutic efficacy of Chinese herbal decoctions in breast cancer treatment (11). For instance, certain decoctions effectively alleviate adverse reactions such as nausea, vomiting, and loss of appetite experienced by breast cancer patients undergoing chemotherapy (12). Furthermore, specific herbal constituents like ginseng and astragalus exhibit potential therapeutic effects in enhancing immune function, exhibiting antitumor activity, and delaying tumor progression (13). However, systematic research and quantitative evidence regarding the mechanisms of action, efficacy assessment, and safety profiles of Chinese herbal decoctions in breast cancer treatment remain insufficient.

Traditional Chinese decoctions are highly diverse, with different formulations possessing distinct combinations of ingredients and therapeutic effects. Commonly used decoctions for breast cancer treatment include formulas that fortify the body’s defenses against pathogens, promote blood circulation and resolve stasis, and clear heat and detoxify (14). These formulations have been clinically employed to alleviate pain, improve appetite, enhance immunity, and inhibit tumor growth in breast cancer patients. Although numerous studies indicate that TCM decoctions possess certain therapeutic efficacy in breast cancer treatment, consistent conclusions remain elusive due to factors such as considerable clinical heterogeneity, non-uniform treatment protocols, and insufficient sample sizes (15, 16). Consequently, conducting a systematic evaluation of the efficacy and safety of different TCM decoctions has become an urgent research priority. This study employs a network meta-analysis methodology to synthesize findings from multiple relevant clinical trials, systematically evaluating the impact of various Chinese herbal decoctions on breast cancer treatment. It aims to provide more reliable supporting evidence for breast cancer patients, thereby offering new therapeutic perspectives and treatment options.

## Methods

This systematic evaluation and meta-analysis were strictly following the PRISMA (Preferred Reporting Items for Systematic Reviews and Meta-Analyses) guidelines (17). And it was registered in Prospero with registration number (CRD420261279914) on 07 January 2026. The review protocol was developed prior to data synthesis, and the registration was completed shortly after the search process as part of protocol formalization and transparency procedures.

### Literature retrieval

A systematic search was conducted in PubMed, Embase, the Cochrane Library, Web of Science, CNKI, Wan fang, and VIP Database, the search period covered the establishment of the databases up to 20 December 2025, The search terms included breast cancer; Chinese herbal decoctions, the specific search strategy is described in **Supplementary Table 1**.

### Inclusion and exclusion criteria

This study will enroll patients diagnosed with breast cancer, with no restrictions on age, gender, ethnicity, or pathological type, provided they are definitively diagnosed with breast cancer. The intervention comprises Chinese herbal decoctions administered as part of adjuvant therapy, which may be used either alone or in combination with conventional treatments such as chemotherapy or radiotherapy. Subjects must be patients receiving Chinese herbal decoction therapy, with the decoction adhering to traditional Chinese medicine principles of syndrome differentiation and treatment, possessing clearly defined medicinal components and administration protocols. The control group was primarily defined as patients receiving chemotherapy alone. Primary outcome measures include objective response rate, disease control rate, CD4+/CD8+ ratio, and nausea and vomiting incidence.

This study will exclude research involving non-breast cancer patients, including those with other malignant tumors or severe comorbidities (such as advanced cancer or organ failure). Studies where the intervention did not involve Chinese herbal decoctions or did not adhere to the principles of TCM syndrome differentiation and treatment will also be excluded. Furthermore, studies lacking reported efficacy or safety data, those with outcome measures not meeting breast cancer treatment standards, and those of low quality, lacking valid data, or featuring poorly designed control groups will be excluded. Literature describing non-clinical research such as non-randomized controlled trials, case reports, or case series will also be excluded.

### Data extractions

Two authors independently screened the literature for inclusion by importing the literature into endnote according to the literature inclusion and exclusion criteria, the final included studies were used for data extraction using excel software and if there was a dispute about the literature screening then it would be discussed, or a third person would be sought to adjudicate. The extracted data contained basic characteristics of the study (author, year of publication), basic characteristics of the population (sample size, gender, age), intervention, and outcome.

### Risk of bias

In the Meta-analysis of this study, we used the ROB 2.0 (Risk of Bias 2.0) tool (18) to assess the risk of bias of the included studies. Developed by the Cochrane Collaboration, the ROB 2.0 tool is a standardized tool for assessing the risk of bias in RCTs, which is aimed at systematically identifying and evaluating bias factors that may affect the validity of the results of the study. This improves the accuracy and reliability of Meta-analyses. The ROB 2.0 tool contains five key assessment domains: randomization process, intervention implementation, outcome measures, data reporting, and other sources of bias. Each domain is scored according to the transparency, reasonableness, and potential for bias in the study design and implementation, and is categorized as low risk, high risk, and uncertain risk. In conducting the assessment, two independent reviewers will score each domain based on the specifics of the study.

### Statistical analysis

In this study, statistical analysis will be performed using a Bayesian framework for Network Meta-Analysis (NMA) (19) to compare the efficacy of Traditional Chinese Medicine Decoctions in breast cancer. First, a treatment network will be constructed by connecting studies that directly or indirectly compare two or more treatments. Each treatment will serve as a node, and edges between nodes represent direct comparisons between treatments performed in individual studies. Next, Bayesian network Meta-analysis was employed, using mean difference (MD) with confidence intervals (95% CrI) as an indicator of treatment effect for continuous outcomes, and odds ratio (OR) 95% CrI for discontinuous outcomes a Bayesian approach allowing the introduction of prior distributions and the use of Markov chain Monte Carlo (MCMC) methods to generate posterior distributions of treatment effects. In this study, local inconsistency assessment using node-splitting models was not performed because the treatment network did not contain closed loops for the analyzed outcomes. Therefore, global consistency was evaluated by comparing the Deviance Information Criterion (DIC) values between consistency and inconsistency models. Specifically, two models will be constructed: one assuming consistency and the other assuming inconsistency. By comparing the DIC values of these two models, we can determine whether there is inconsistency in the network. To account for inter-study heterogeneity, a random effects model will be used for the analysis. Heterogeneity will be assessed by the I² statistic, with significant heterogeneity indicated if the I² value exceeds 50%. Network consistency will also be tested to ensure consistency between direct and indirect evidence. All statistical analyses will be performed using R software (version 4.0.0) and the “gemtc” package, which is specifically designed for Bayesian network meta-analysis. The ranking probability of each intervention was summarized using the Surface Under the Cumulative Ranking curve (SUCRA).

## Results

### Literature retrieval results

**Figure 1:**
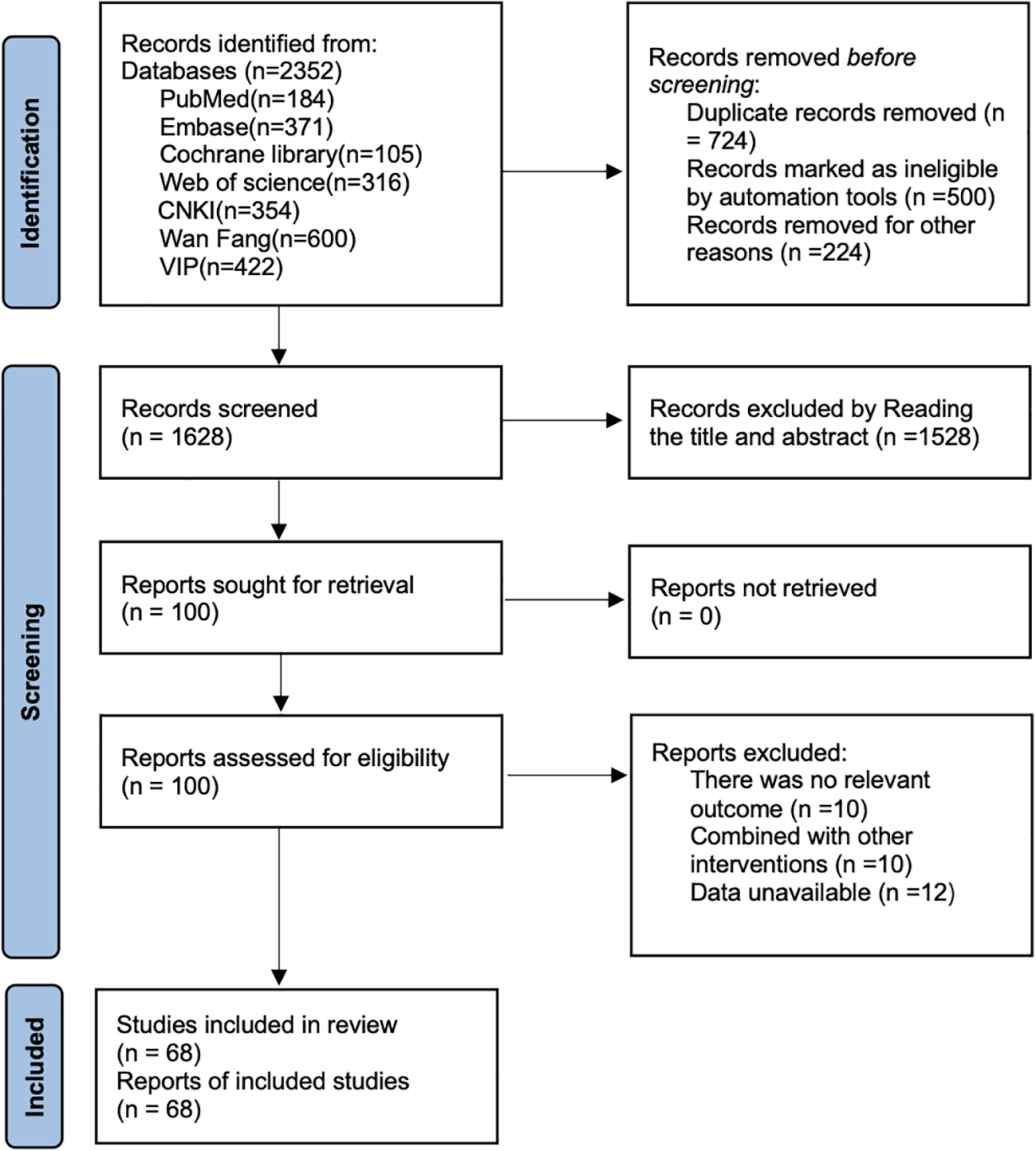
Literature search flow chart.

As shown in **Figure 1**, searches of PubMed (n=184), Embase (n=371), the Cochrane Library (n=105), Web of Science (n=316), CNKI (n=354), Wan fang (n=600), and VIP (n=422) yielded a total of 2,352 articles. After removing 724 duplicate records, 1,528 articles were screened based on abstracts and titles. Following full-text review, 32 articles were excluded, resulting in 68 studies ultimately included for analysis.

### Basic characteristics of the included studies

This study included a total of 68 articles involving 4,708 patients. The interventions comprised 14 decoctions: Bazhen Decoction (BZT), Bushen Huoxue Decoction (BSHXT), Chaihu Jialonggu Muli Decoction (CHJLGMLT), Fangji Huangqi Decoction (FJHQT), Fuzheng Xiaoliu Decoction (FZXLT), Guipi Decoction (GPT), Huangqi Jiedu Decoction (HQJDT), Ruyan Decoction (RYT), Taohong Siwu Decoction (THSWT), Xiangsha Liujunzi Decoction (XSLJZT), Xiaoaishunqi Decoction (XASQT), Xiaochaihu Decoction (XCHT), Yanghe Huayan Decoction (YHHYT), and Yanghe Decoction (YHT). Specific fundamental characteristics are detailed in **Supplementary Table 2**, whilst the composition of each decoction is outlined in **Supplementary Table 3**.

### Risk of bias results

This study employed Risk of Bias 2.0 for quality assessment. Results (**Supplementary Figure 1**, **Supplementary Table 4**) indicate that 34 studies were judged as having a low risk of bias, and 34 studies were judged as having some concerns. For the randomization process, 64 studies were rated as low risk and 4 as having some concerns. For deviations from intended interventions, 48 studies were rated as low risk and 20 as having some concerns. For missing outcome data, 58 studies were rated as low risk and 10 as having some concerns. For measurement of the outcome, 52 studies were rated as low risk and 16 as having some concerns. For selection of the reported result, 56 studies were rated as low risk and 12 as having some concerns. No study was judged as having a high overall risk of bias. The main methodological concerns were related to incomplete reporting of intervention implementation, outcome measurement, and selective reporting in some trials. Overall, the methodological quality of the included studies was considered moderate.

### Consistency test results

Because the treatment networks mainly exhibited a star-shaped structure without closed loops, local inconsistency analyses such as node-splitting could not be conducted. Therefore, global consistency was assessed using the DIC values of consistency and inconsistency models, results (**Supplementary Table 5**) indicate that the objective response rate, disease control rate, CD4+/CD8+ ratio, and nausea and vomiting all demonstrate consistency.

### Pairwise meta- analysis results

Pairwise meta-analysis and heterogeneity assessment were performed for all direct comparisons, and the detailed results are presented in **Table 1**. For the objective response rate, heterogeneity was generally low, with I² values ranging from 0% to 36.2%. Significant improvements were observed for FZXLT versus Control [OR = 2.00, 95%CrI (1.06, 3.80), I² = 0%], HQJDT versus Control [OR = 2.45, 95%CrI (1.56, 3.90), I² = 0%], RYT versus Control [OR = 3.14, 95%CrI (1.61, 6.46), I² = 0%], XASQT versus Control [OR = 3.10, 95%CrI (1.98, 4.96), I² = 36.2%], XCHT versus Control [OR = 2.47, 95%CrI (1.47, 4.09), I² = 0%], YHHYT versus Control [OR = 4.72, 95%CrI (1.15, 25.79)], and YHT versus Control [OR = 2.02, 95%CrI (1.18, 3.43), I² = 0%].

**Table 1:** Results of pairwise meta-analysis.

Outcomes	Pairwise meta-analysis	No of study	Heterogeneity I^2^ (%)	OR 95%CrI
Objective response rate	Control vs BSHXT	1	NA	0.56 (0.20,1.51)
	Control vs BZT	2	0	0.47 (0.21,1.01)
	FJHQT vs Control	1	NA	2.28 (0.92,5.43)
	FZXLT vs Control	2	0	2.00 (1.06,3.80)
	HQJDT vs Control	4	0	2.45 (1.56,3.90)
	RYT vs Control	3	0	3.14 (1.61,6.46)
	THSWT vs Control	1	NA	5.09 (0.92,60.46)
	XASQT vs Control	6	36.2	3.10 (1.98,4.96)
	XCHT vs Control	4	0	2.47 (1.47,4.09)
	XSLJZT vs Control	1	NA	1.73 (0.75,4.02)
	YHHYT vs Control	1	NA	4.72 (1.15,25.79)
	YHT vs Control	6	0	2.02 (1.18,3.43)
Outcomes	Pairwise meta-analysis	No of study	Heterogeneity (%)	OR 95%CrI
Disease control rate	Control vs BSHXT	1	NA	0.22 (0.03,1.28)
	Control vs BZT	1	NA	0.16 (0.02,0.92)
	FJHQT vs Control	1	NA	2.83 (0.64,12.34)
	FZXLT vs Control	2	80.3	0.94 (0.34,3.13)
	HQJDT vs Control	3	22.6	4.35 (1.76,12.63)
	RYT vs Control	3	4.5	2.97 (1.02,9.94)
	XASQT vs Control	5	0	4.12 (1.67,11.27)
	XCHT vs Control	4	0	4.44 (1.88,11.07)
	XSLJZT vs Control	1	NA	5.29 (0.78,53.91)
	YHHYT vs Control	1	NA	3.89 (0.53,40.23)
	YHT vs Control	5	24	2.68 (1.10,7.08)
CD4+/CD8+	Control vs BSHXT	2	58.8	-0.44 (-0.89,0.01)
	Control vs BZT	2	99.6	-0.32 (-0.77,0.13)
	FJHQT vs Control	2	70.4	0.69 (0.20,1.17)
	FZXLT vs Control	7	69.8	0.25 (0.01,0.50)
	GPT vs Control	3	93.5	0.42 (0.04,0.80)
	RYT vs Control	3	87.6	0.55 (0.17,0.93)
	XASQT vs Control	7	99.1	-0.04 (-0.28,0.21)
	XCHT vs Control	1	NA	0.34 (-0.29,0.98)
	XSLJZT vs Control	2	73.2	0.18 (-0.28,0.64)
	YHHYT vs Control	2	97	0.07 (-0.39,0.55)
	YHT s Control	2	92.8	0.27 (-0.18,0.72)
Nausea and vomiting	Control vs BSHXT	3	62.4	2.69(1.10, 6.71)
	Control vs BZT	3	0	3.82(1.32, 11.98)
	Control vs CHJLGMLT	2	0	4.28(0.78,32.07)
	FJHQT vs Control	6	0	0.27 (0.10,0.68)
	FZXLT vs Control	1	NA	1.01 (0.26,4.04)
	GPT vs Control	1	NA	0.26(0.03,1.85)
	HQJDT vs Control	1	NA	0.71 (0.09,4.82)
	RYT vs Control	2	68.9	0.41 (0.10,1.65)
	XCHT vs Control	4	0	0.70 (0.28,1.71)
	XSLJZT vs Control	3	10.5	0.47 (0.16,1.15)
	YHHYT vs Control	1	NA	0.32(0.08,1.25)
	YHT vs Control	5	50.4	0.29 (0.14,0.60)

BZT, Bazhen Decoction; BSHXT, Bushen Huoxue Decoction; CHJLGMLT, Chaihu Jialonggu Muli Decoction; FJHQT, Fangji Huangqi Decoction; FZXLT, Fuzheng Xiaoliu Decoction; GPT, Guipi Decoction; HQJDT, Huangqi Jiedu Decoction; RYT, Ruyan Decoction; THSWT, Taohong Siwu Decoction; XSLJZT, Xiangsha Liujunzi decoction; XASQT, Xiaoaishunqi Decoction; XCHT, Xiaochaihu Decoction; YHHYT, Yanghe Huayan Decoction; YHT, Yanghe Decoction; NR, not reported.

For the disease control rate, most comparisons showed low-to-moderate heterogeneity, although substantial heterogeneity was observed for FZXLT versus Control [I² = 80.3%]. Significant improvements were observed for HQJDT versus Control [OR = 4.35, 95%CrI (1.76, 12.63), I² = 22.6%], RYT versus Control [OR = 2.97, 95%CrI (1.02, 9.94), I² = 4.5%], XASQT versus Control [OR = 4.12, 95%CrI (1.67, 11.27), I² = 0%], XCHT versus Control [OR = 4.44, 95%CrI (1.88, 11.07), I² = 0%], and YHT versus Control [OR = 2.68, 95%CrI (1.10, 7.08), I² = 24.0%].

For CD4+/CD8+, several comparisons demonstrated substantial heterogeneity, including BSHXT versus Control [I² = 58.8%], BZT versus Control [I² = 99.6%], FJHQT versus Control [I² = 70.4%], FZXLT versus Control [I² = 69.8%], GPT versus Control [I² = 93.5%], RYT versus Control [I² = 87.6%], XASQT versus Control [I² = 99.1%], XSLJZT versus Control [I² = 73.2%], YHHYT versus Control [I² = 97.0%], and YHT versus Control [I² = 92.8%]. Nevertheless, significant improvements were still observed for FJHQT versus Control [MD = 0.69, 95%CrI (0.20, 1.17)], FZXLT versus Control [MD = 0.25, 95%CrI (0.01, 0.50)], GPT versus Control [MD = 0.42, 95%CrI (0.04, 0.80)], and RYT versus Control [MD = 0.55, 95%CrI (0.17, 0.93)].

For nausea and vomiting, heterogeneity ranged from 0% to 68.9%. Moderate-to-substantial heterogeneity was observed for BSHXT versus Control [I² = 62.4%], RYT versus Control [I² = 68.9%], and YHT versus Control [I² = 50.4%]. Significant reductions in nausea and vomiting were observed for BSHXT versus Control [OR = 2.69, 95%CrI (1.10, 6.71)], BZT versus Control [OR = 3.82, 95%CrI (1.32, 11.98)], FJHQT versus Control [OR = 0.27, 95%CrI (0.10, 0.68), I² = 0%], and YHT versus Control [OR = 0.29, 95%CrI (0.14, 0.60), I² = 50.4%].

whereas the corresponding network meta-analysis estimates are reported in the league tables in **Supplementary Tables 6**–**S9**.

### Objective response rate

**Figure 2:**
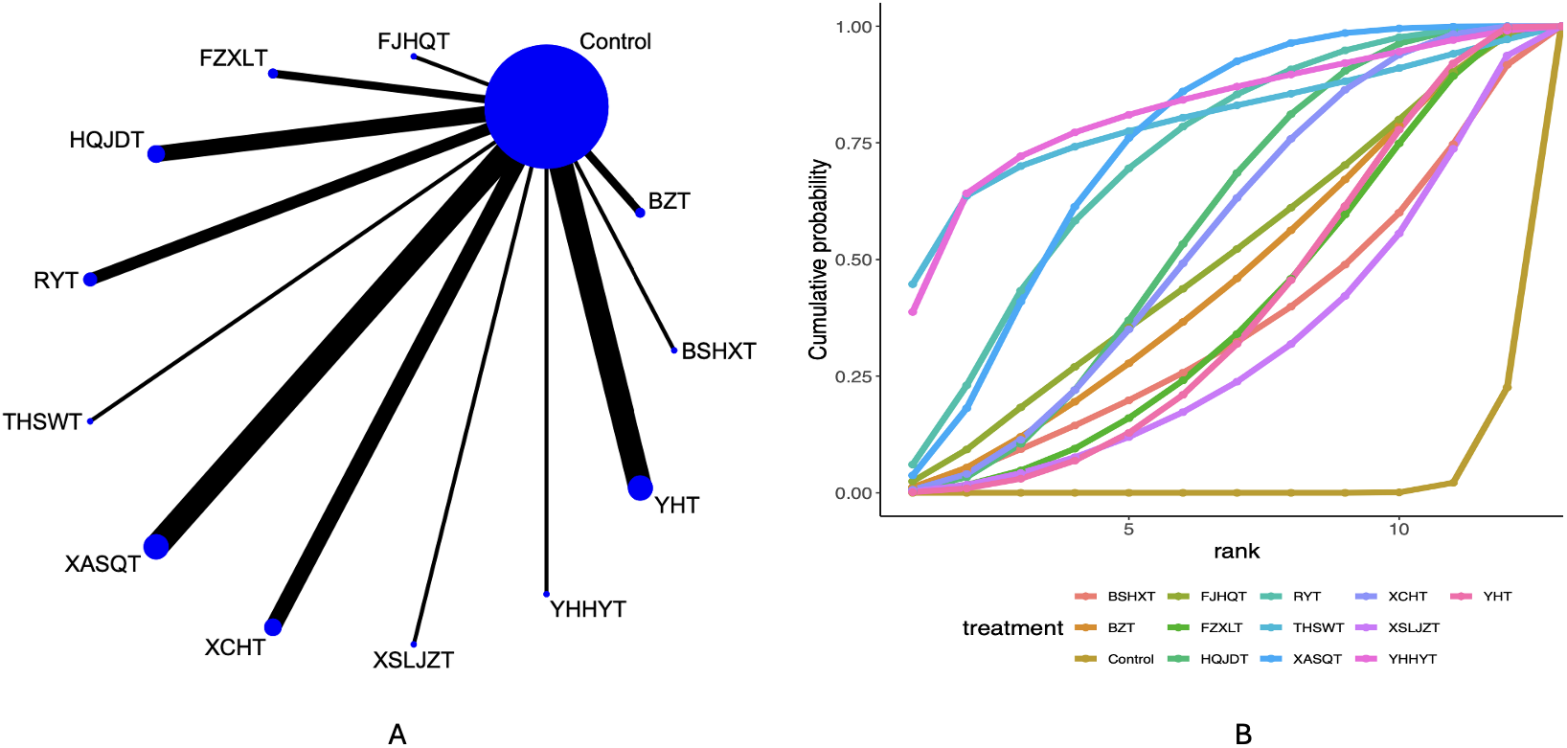
Results of meta-analysis of objective response rate [**(A)** network plot, **(B)** rank under cumulative probability curve].

Thirty-two articles mentioned the objective response rate, with the network diagram (**Figure 2A**) indicating that BZT, BSHXT, FJHQT, FZXLT, HQJDT, RYT, THSWT, XASQT, XCHT, XSLJZT, YHHYT, and YHT formed direct comparisons with the control. The league table (**Supplementary Table 6**) indicates that compared with control, BZT [OR = 2.15, 95%CrI (1.06, 4.42)], FJHQT [OR = 2.27, 95%CrI (1.03, 5.00)], FZXLT [OR = 2.00, 95%CrI (1.12, 3.57)], HQJDT [OR = 2.5, 95%CrI (1.64, 3.70)], RYT [OR = 3.13, 95%CrI (1.67, 5.88)], XASQT [OR = 3.13, 95%CrI (2.00, 4.76)], YHHYT[OR = 4.76, 95%CrI (1.25, 25)] demonstrated the potential to enhance the objective response rate in breast cancer patients, though no statistically significant differences were observed between the various decoctions. The SUCRA ranking (**Table 2**, **Figure 2B**) indicated YHHYT (81.42%) > THSWT (79.13%) > XASQT (72.76%) > Control (2.07%).

**Table 2:** SUCRA ranking.

Treatment	Objective response rate (%)	Disease control rate (%)	CD4/CD8 (%)	Nausea and vomiting (%)
BSHXT	35.22	65.02	67.69	58.19
BZT	44.94	74.66	54.68	75.67
CHJLGMLT	NR	NR	NR	72.29
Control	2.07	7.02	14.52	9.77
FJHQT	48.99	44.43	88.00	72.30
FZXL	38.28	4.50	46.75	12.65
HQJDT	55.18	62.38	NR	9.77
GPT	NR	NR	66.18	68.29
RYT	70.55	43.52	79.78	53.79
THSWT	79.13	NR	NR	NR
XASQT	72.76	63.76	12.47	NR
XCHT	53.33	66.65	55.58	25.32
XSLJZT	30.34	68.89	38.74	38.66
YHHYT	81.42	59.54	26.64	64.16
YHT	37.78	39.61	48.95	67.76

BZT, Bazhen Decoction; BSHXT, Bushen Huoxue Decoction; CHJLGMLT, Chaihu Jialonggu Muli Decoction; FJHQT, Fangji Huangqi Decoction; FZXLT, Fuzheng Xiaoliu Decoction; GPT, Guipi Decoction; HQJDT, Huangqi Jiedu Decoction; RYT, Ruyan Decoction; THSWT, Taohong Siwu Decoction; XSLJZT, Xiangsha Liujunzi decoction; XASQT, Xiaoaishunqi Decoction; XCHT, Xiaochaihu Decoction; YHHYT, Yanghe Huayan Decoction; YHT, Yanghe Decoction; NR, not reported.

### Disease control rate

**Figure 3:**
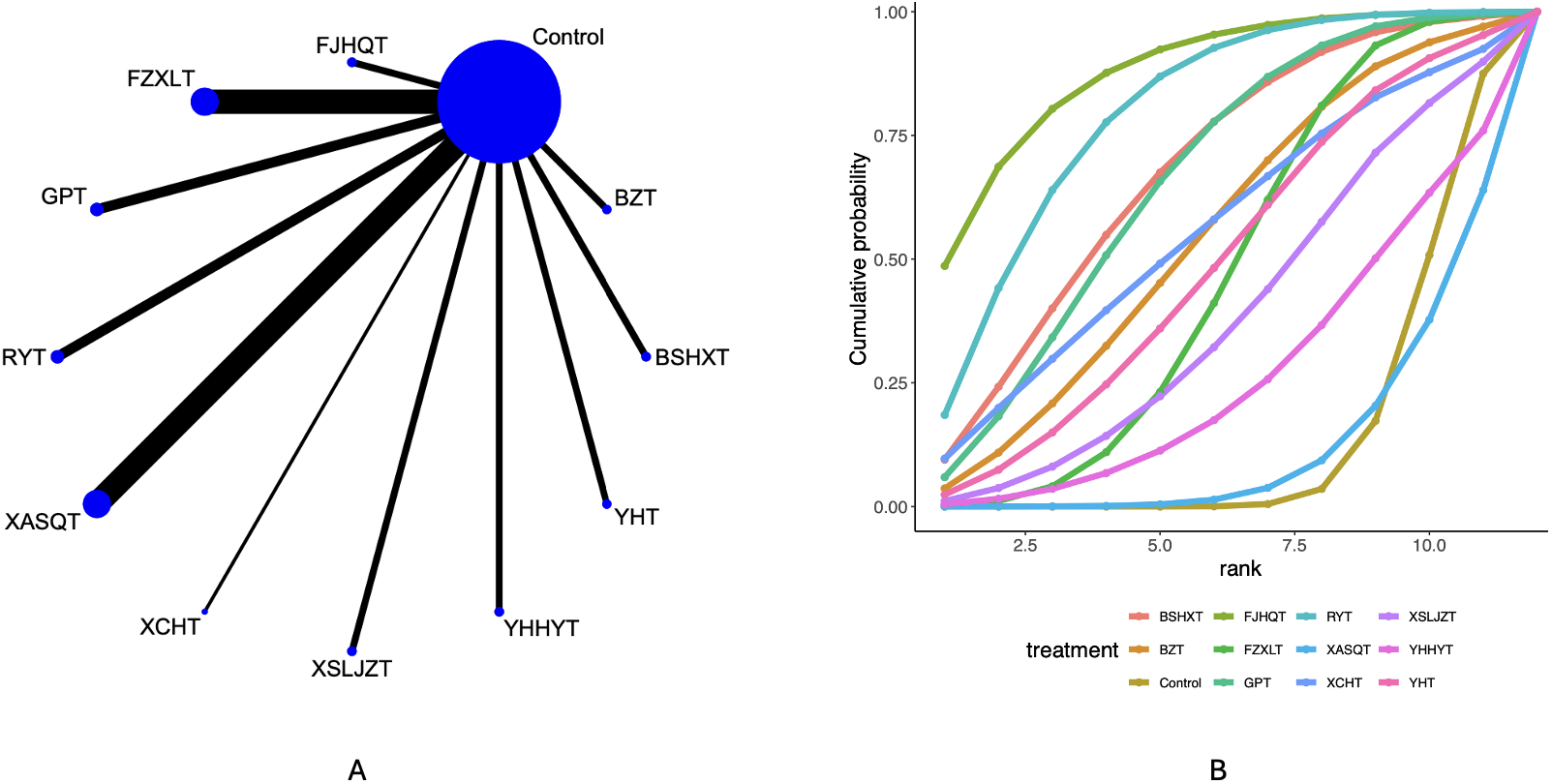
Results of meta-analysis of disease control rate [**(A)** network plot, **(B)** rank under cumulative probability curve].

Twenty-six articles mentioned the disease control rate, with the network diagram (**Figure 3A**) indicating that BZT, BSHXT, FJHQT, FZXLT, HQJDT, RYT, THSWT, XASQT, XCHT, XSLJZT, YHHYT, and YHT formed direct comparisons with the control. The league table (**Supplementary Table 7**) indicates that compared with control, BZT [OR = 5.77, 95%CrI (1.59, 29)], FJHQT [OR = 2.78, 95%CrI (1.08, 7.69)], HQJDT [OR = 4.00, 95%CrI (2.04, 8.33)], RYT [OR = 2.78, 95%CrI (1.16, 6.67)], XASQT [OR = 4.17, 95%CrI (1.96, 10.00)], XCHT [OR = 4.35, 95%CrI (2.33, 8.33)], XSLJZT[OR = 5.00, 95%CrI (1.14, 33.33)], YHT[OR = 2.56, 95%CrI (1.28, 5.56)] demonstrated the potential to enhance the disease control rate in breast cancer patients, Moreover, BZT [OR = 6.79, 95%CrI (1.52, 39.73)] and BSHXT [OR = 5.27, 95%CrI (1.15, 31.93)] demonstrated superior efficacy compared to FZXLT. The SUCRA ranking (**Table 2**, **Figure 3B**) indicated BZT (74.66%) > XSLJZT (68.89%) > XCHT (66.65%) > Control (7.02%).

### CD4+/CD8+

**Figure 4:**
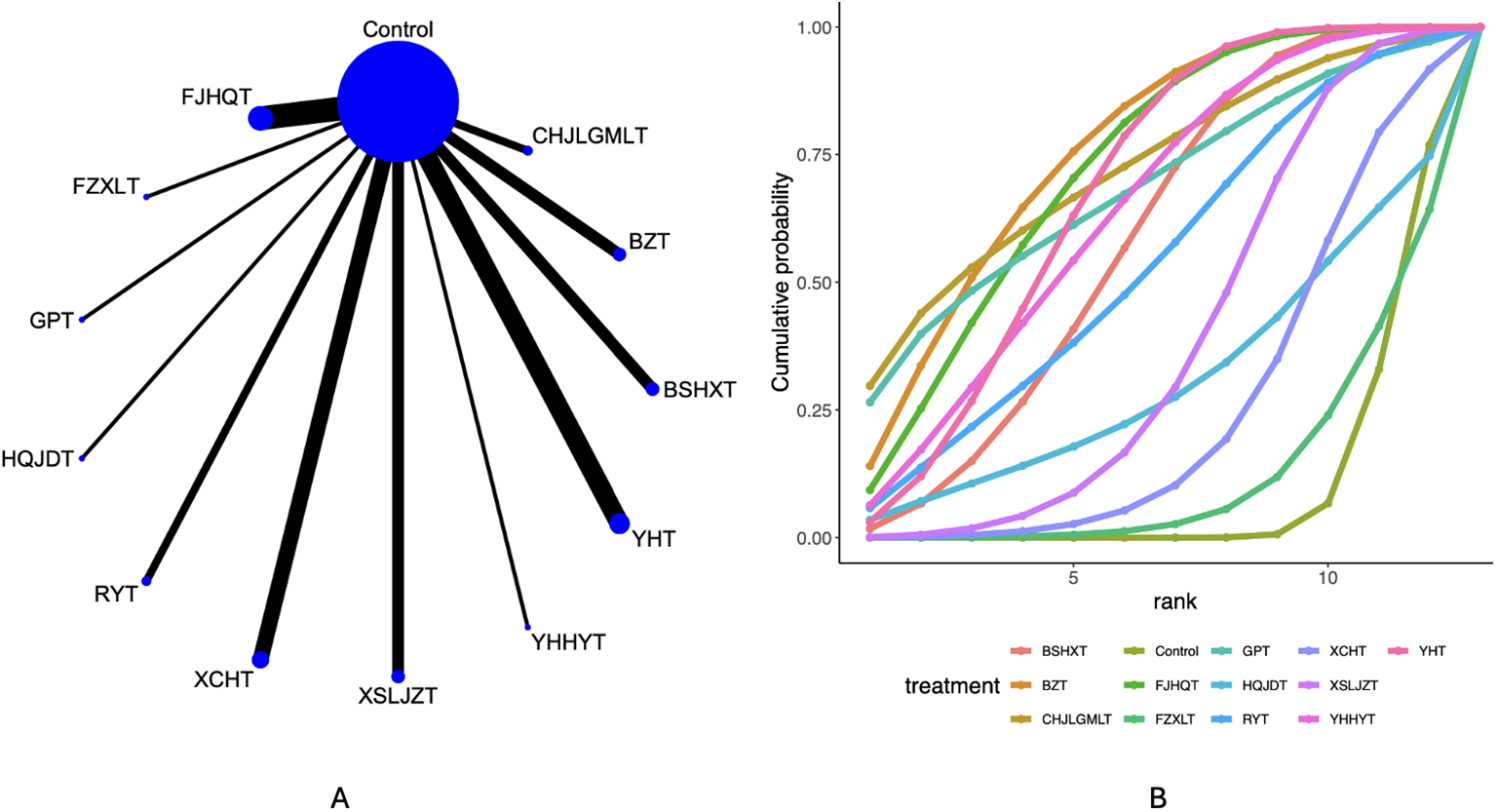
Results of meta-analysis of CD4+/CD8+ [**(A)** network plot, **(B)** rank under cumulative probability curve].

Thirty-three articles mentioned the CD4+/CD8+, with the network diagram (**Figure 4A**) indicating that BZT, BSHXT, FJHQT, FZXLT, GPT, RYT, XASQT, XCHT, XSLJZT, YHHYT, and YHT formed direct comparisons with the control. The league table (**Supplementary Table 8**) indicates that compared with control, FJHQT [MD = 0.69, 95%CrI (0.2, 1.17)], GPT [MD = 0.42, 95%CrI (0.04,0.8)], RYT [MD = 0.55, 95%CrI (0.18, 0.93)] demonstrated the potential to enhance the CD4+/CD8+ in breast cancer patients, Moreover, RYT demonstrated superior efficacy compared to FZXLT[MD = 0.59, 95%CrI (0.14, 1.03)]. The SUCRA ranking (**Table 2**, **Figure 4B**) indicated FJHQT (88.00%)>RYT (79.78%) > BSHXT (67.69%) > Control (14.52%).

### Nausea and vomiting

**Figure 5:**
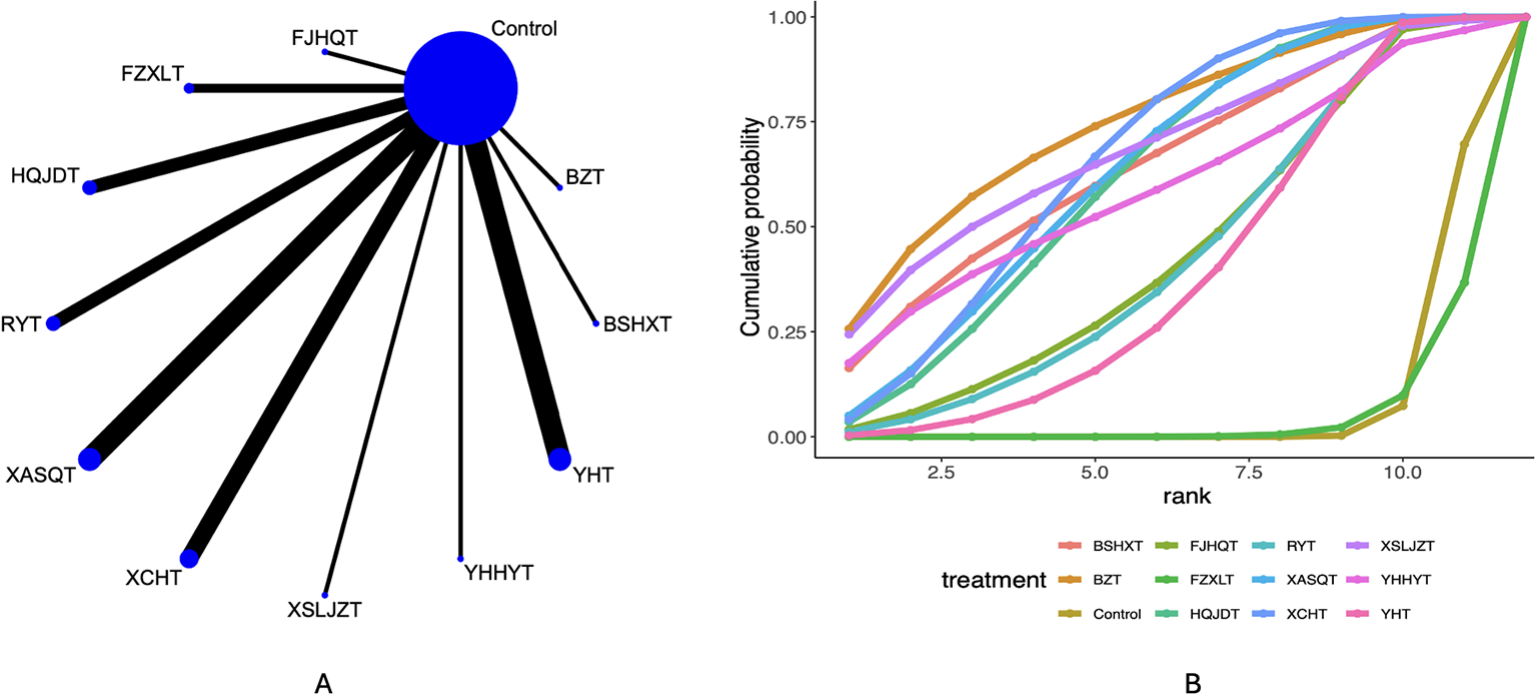
Results of meta-analysis of nausea and vomiting [**(A)** network plot, **(B)** rank under cumulative probability curve].

Thirty-three articles mentioned the nausea and vomiting, with the network diagram (**Figure 5A**) indicating that BSHXT, BZT, CHJLGMLT, FJHQT, FZXL, HQJDT, GPT, RYT, XCHT, XSLJZT, YHHYT and YHT formed direct comparisons with the control. The league table (**Supplementary Table 9**) indicates that compared with control, BSHXT [OR = 0.36, 95%CrI (0.19, 0.69)], BZT [OR = 0.25, 95%CrI (0.1, 0.57)], FJHQT [OR = 0.27, 95%CrI (0.12, 0.57)], XSLJZT[OR = 0.52, 95%CrI (0.28, 0.97)], YHHYT [OR = 0.32, 95%CrI (0.13, 0.76)], YHT[OR = 0.30, 95%CrI (0.17, 0.57))] demonstrated the potential to reduce the nausea and vomiting in breast cancer patients, Moreover, BZT [OR = 0.25, 95%CrI (0.07, 0.8)] and FJHQT [OR = 0.27, 95%CrI (0.08, 0.82)] demonstrated superior efficacy compared to FZXLT. The SUCRA ranking (**Table 2**, **Figure 5B**) indicated BZT (75.67%) > FJHQT (72.30%) > CHJLGMLT (72.29%) > Control (9.77%).

### Publication bias

This study employed funnel plots to assess publication bias. The results (**Supplementary Figures 2**–**S5**) indicate that the objective response rate, disease control rate, CD4+/CD8+ ratio, and nausea and vomiting funnel plots exhibit symmetry, suggesting a low likelihood of publication bias.

## Discussion

This network meta-analysis evaluated the comparative efficacy and safety of different traditional Chinese medicine decoctions combined with chemotherapy for breast cancer. The results suggested that several decoctions may provide additional benefits when used as adjunctive therapies to chemotherapy, particularly in improving objective response rate, disease control rate, and CD4+/CD8+ ratio, as well as reducing nausea and vomiting. According to the SUCRA rankings, YHHYT ranked highest for objective response rate, BZT ranked highest for disease control rate and nausea and vomiting, and FJHQT ranked highest for CD4+/CD8+. However, these rankings should be interpreted cautiously because SUCRA reflects relative ranking probabilities rather than definitive clinical superiority, and the certainty of evidence may be affected by study quality, sample size, heterogeneity, and the lack of direct head-to-head comparisons between different decoctions.

The potential clinical benefits of Chinese herbal decoctions may be related to their adjunctive role during chemotherapy. In the included studies, the control group mainly received chemotherapy alone, whereas the experimental groups received specific decoctions in combination with chemotherapy (20). Therefore, the findings should be interpreted as the additional effect of decoctions on the basis of chemotherapy rather than as evidence that decoctions can replace standard anticancer treatment. This distinction is important for clinical interpretation (21). The observed improvements in tumor response and disease control may suggest a potential supportive role of decoctions in comprehensive breast cancer management, but further high-quality randomized controlled trials are needed before firm recommendations can be made.

Previous experimental and pharmacological studies (22, 23) have suggested that some decoctions may exert immunomodulatory, anti-inflammatory, antioxidant, or tumor microenvironment–regulating effects. These mechanisms may partly explain the clinical trends observed in this study. For example, previous studies have proposed that YHHYT may influence tumor-related immune responses and pathways associated with tumor progression. Similarly, BZT and FJHQT have been discussed in prior literature as formulas with potential effects on immune regulation, inflammatory responses, and treatment-related adverse reactions (24, 25). Nevertheless, the present network meta-analysis did not directly assess molecular pathways, pharmacological targets, or mechanistic biomarkers. Therefore, these mechanistic explanations should be regarded as hypotheses derived from previous literature rather than direct evidence generated by the present study (26).

The improvement in CD4+/CD8+ ratio observed for some decoctions may indicate a potential effect on immune status during chemotherapy. However, substantial heterogeneity was observed in several pairwise comparisons for this outcome. This heterogeneity may be attributable to differences in chemotherapy regimens, treatment duration, decoction composition and dosage, tumor stage, baseline immune function, and measurement methods (27, 28). Therefore, although the CD4+/CD8+ findings are clinically relevant, they should be interpreted with caution. Future studies should standardize immune outcome measurement and report baseline immune indicators more completely.

Nausea and vomiting are common chemotherapy-related adverse events that can impair treatment adherence and quality of life. In this study, several decoctions appeared to reduce nausea and vomiting compared with chemotherapy alone (29). This finding suggests that Chinese herbal decoctions may have a supportive role in managing chemotherapy-related gastrointestinal toxicity (30). However, differences in chemotherapy regimens, antiemetic use, symptom grading, and follow-up duration across studies may have influenced the pooled estimates. Future trials should adopt standardized adverse event grading systems and clearly report concomitant supportive care (31, 32).

### Contribution to the literature

This study contributes to the existing literature by providing a comparative synthesis of multiple Chinese herbal decoctions combined with chemotherapy for breast cancer. Unlike conventional pairwise meta-analyses focusing on single decoctions, this network meta-analysis integrates direct and indirect evidence to compare several decoction-based regimens within one analytical framework (33). The findings provide preliminary ranking information regarding efficacy and safety outcomes, which may help identify potentially promising decoctions for future high-quality randomized controlled trials and inform evidence-based adjunctive treatment strategies.

### Strengths and limitations

This study has several strengths. First, it synthesized evidence from 68 randomized controlled trials and compared multiple decoctions using a network meta-analysis framework. Second, both efficacy and safety-related outcomes were considered, providing a broader evaluation of the potential adjunctive value of decoctions in breast cancer treatment. Third, consistency was assessed by comparing the DIC values between consistency and inconsistency models. Because the treatment networks mainly showed a star-shaped structure without closed loops, local inconsistency tests such as node-splitting could not be performed. The absence of closed loops also reflects the current lack of direct head-to-head trials comparing different decoctions.

Several limitations should also be acknowledged. First, although all included studies were randomized controlled trials, the overall methodological quality varied. Some studies had concerns related to deviations from intended interventions, outcome measurement, or selective reporting, mainly due to insufficient reporting of allocation concealment, blinding procedures, and protocol details (34). Second, the sample sizes of several included studies were relatively small, which may have reduced statistical precision and increased the risk of unstable estimates (35). Third, although the control groups mainly consisted of chemotherapy alone, the specific chemotherapy regimens, treatment cycles, supportive care strategies, and decoction compositions were not completely identical across studies, potentially contributing to clinical heterogeneity.

Fourth, substantial heterogeneity was observed in several pairwise comparisons, particularly for CD4+/CD8+ outcomes (36). Differences in patient characteristics, tumor stage, baseline immune status, treatment duration, outcome measurement methods, and herbal formulation modifications may partly explain this heterogeneity. Therefore, some pooled estimates should be interpreted cautiously. Fifth, most treatment networks exhibited a star-shaped structure in which different decoctions were primarily compared with chemotherapy alone, while direct head-to-head comparisons between different decoctions were limited. Consequently, closed loops were not formed for most outcomes, preventing local inconsistency assessment using node-splitting models. Although global consistency assessed by DIC suggested acceptable overall consistency, the lack of closed-loop evidence remains an inherent limitation of the available literature.

Sixth, the SUCRA rankings provide relative ranking probabilities rather than definitive evidence of clinical superiority. Rankings may be unstable when evidence is sparse, heterogeneous, or imprecise, and should therefore be interpreted cautiously. Seventh, the present network meta-analysis evaluated clinical outcomes only and did not directly investigate molecular or pharmacological mechanisms. Mechanistic interpretations discussed in this manuscript were derived from previous experimental and pharmacological studies rather than directly validated by the present analysis. Finally, most included studies were conducted in China, which may limit the generalizability of the findings to other populations and healthcare settings. Future large-scale, multicenter, rigorously designed randomized controlled trials with standardized chemotherapy protocols, outcome definitions, and long-term follow-up are required to further validate the comparative efficacy and safety of these decoction-based interventions.

## Conclusion

This study employed a network meta-analysis to evaluate the efficacy of multiple Chinese herbal decoctions in breast cancer treatment. Results demonstrated that these decoctions exhibited significant therapeutic effects in enhancing target response rates, disease control rates, and immune function (CD4+/CD8+ ratio) among breast cancer patients, while also alleviating chemotherapy side effects such as nausea and vomiting. Although no statistically significant differences in efficacy were observed between the various decoctions, their multi-targeted actions and modulation of the tumor immune microenvironment suggest they hold promise as important adjuvant therapies in breast cancer treatment. Future high-quality randomized controlled trials are required to further validate the long-term efficacy and safety of these decoctions, as well as their combined application with modern anti-cancer treatments, thereby providing breast cancer patients with more personalized therapeutic options.

## Data Availability

The original contributions presented in the study are included in the article/**Supplementary Material**. Further inquiries can be directed to the corresponding author.

## References

[1] Zhao L Yuan J Yang Q Ma J Yang F Zou Y . Diabetes and its complications: molecular mechanisms, prevention and treatment. Signal Transduct Target Ther. (2026) 11(1):22. doi: 10.1038/s41392-025-02401-w 41549124 PMC12812840

[2] Martinez Leon V Hilburg R Susztak K . Mechanisms of diabetic kidney disease and established and emerging treatments. Nat Rev Endocrinol. (2026) 22(1):21–35. doi: 10.1038/s41574-025-01171-3 40935879 PMC13714763

[3] Li X He JC Lee K . New potential therapeutic targets of metabolic disorder-associated kidney disease and diabetic kidney disease. Kidney Int. (2026) 109(1):81–8. doi: 10.1016/j.kint.2025.09.023 41139023 PMC13470854

[4] Zhao H Guo J . Macrophages in Focus: Key Drivers and Therapeutic Opportunities in Diabetic Kidney Disease. Int J Biol Sci. (2025) 21(10):4647–62. doi: 10.7150/ijbs.112737 40765824 PMC12320494

[5] Fan X Yang M Lang Y Lu S Kong Z Gao Y . Mitochondrial metabolic reprogramming in diabetic kidney disease. Cell Death Dis. (2024) 15(6):442. doi: 10.1038/s41419-024-06833-0 38910210 PMC11194272

[6] Natale P Palmer SC Navaneethan SD Craig JC Strippoli GF . Angiotensin-converting-enzyme inhibitors and angiotensin receptor blockers for preventing the progression of diabetic kidney disease. Cochrane Database Syst Rev. (2024) 4(4):Cd006257. 38682786 10.1002/14651858.CD006257.pub2 PMC11057222

[7] van Raalte DH Bjornstad P Cherney DZI de Boer IH Fioretto P Gordin D . Combination therapy for kidney disease in people with diabetes mellitus. Nat Rev Nephrol. (2024) 20(7):433–46. doi: 10.1038/s41581-024-00827-z 38570632

[8] Yi TW Sridhar VS Scott J Nardone M Cherney D . Next-generation therapeutics for diabetic kidney disease. Nat Rev Nephrol. (2026). doi: 10.1038/s41581-025-01042-0 41526484

[9] Huo JL Li P Feng Q Fu W Pan S Liu D . Targeting Mitochondrial Dysfunction by Natural Products for the Treatment of Diabetic Kidney Disease. Phytother Res. (2025) 39(12):5717–43. doi: 10.1002/ptr.70059 40738853

[10] Wu XQ Zhao L Zhao YL He XY Zou L Zhao YY . Traditional Chinese medicine improved diabetic kidney disease through targeting gut microbiota. Pharm Biol. (2024) 62(1):423–35. doi: 10.1080/13880209.2024.2351946 38757785 PMC11104709

[11] Shen S Zhong H Zhou X Li G Zhang C Zhu Y . Advances in Traditional Chinese Medicine research in diabetic kidney disease treatment. Pharm Biol. (2024) 62(1):222–32. doi: 10.1080/13880209.2024.2314705 38357845 PMC10877659

[12] Liu XJ Hu XK Yang H Gui LM Cai ZX Qi MS . A Review of Traditional Chinese Medicine on Treatment of Diabetic Nephropathy and the Involved Mechanisms. Am J Chin Med. (2022) 50(7):1739–79. doi: 10.1142/S0192415X22500744 36222120

[13] Chen Y Huang G Qin T Zhang Z Wang H Xu Y . Ferroptosis: A new view on the prevention and treatment of diabetic kidney disease with traditional Chinese medicine. BioMed Pharmacother. (2024) 170:115952. doi: 10.1016/j.biopha.2023.115952 38056233

[14] Li H Chen H Gao R Yin M Huang F . Traditional Chinese Medicine Formulae and Chinese Patent Medicines for the Treatment of Diabetic Kidney Disease: Efficacies and Mechanisms. Am J Chin Med. (2025) 53(3):675–707. doi: 10.1142/S0192415X25500260 40374376

[15] Sheng L Cao Z Wang L Xu Y Gui D . Research progress in the treatment of lipid metabolism disorder in patients with diabetic kidney disease by the integrated traditional Chinese and Western medicine. Front Endocrinol (Lausanne). (2025) 16:1631312. doi: 10.3389/fendo.2025.1631312 40810065 PMC12343256

[16] Li P Zheng H Ma J Lu W Li L Liu F . Impact of finerenone on chronic kidney disease progression in Chinese patients with type 2 diabetes: a FIGARO-DKD subgroup analysis. Front Endocrinol (Lausanne). (2025) 16:1568438. doi: 10.3389/fendo.2025.1568438 40370775 PMC12074935

[17] Liu J Gao LD Fu B Yang HT Zhang L Che SQ . Efficacy and safety of Zicuiyin decoction on diabetic kidney disease: A multicenter, randomized controlled trial. Phytomedicine. (2022) 100:154079. doi: 10.1016/j.phymed.2022.154079 35413644

[18] Page MJ McKenzie JE Bossuyt PM Boutron I Hoffmann TC Mulrow CD . The PRISMA 2020 statement: an updated guideline for reporting systematic reviews. Bmj. (2021) 372:n71. 33782057 10.1136/bmj.n71 PMC8005924

[19] Lu T Lu C Li H Xing X Deng X Li X . The reporting quality and risk of bias of randomized controlled trials of acupuncture for migraine: Methodological study based on STRICTA and RoB 2.0. Complement Ther Med. (2020) 52:102433. doi: 10.1016/j.ctim.2020.102433 32951707

[20] Guyatt GH Oxman AD Vist GE Kunz R Falck-Ytter Y Alonso-Coello P . GRADE: an emerging consensus on rating quality of evidence and strength of recommendations. Bmj. (2008) 336(7650):924–6. doi: 10.1136/bmj.39489.470347.AD 18436948 PMC2335261

[21] Zhen B Chen H Maimaitiyasen D Li X Xiao H Li X . Analysis of Treatment of Diabetic Kidney Disease with Modified Buyang Huanwutang Based on 5hmC-Seal Sequencing Technology. Chin J Exp Traditional Med Formulae. (2025) 31(11):208–17.

[22] Wang N Feng H Zhang Z Tian H Gu L Bian Y . Danggui Buxue decoction regulates autophagy to Improve renal fibrosis in diabetes through miR-27a /PI3K/AKT pathway. J Ethnopharmacol. (2025) 341:119357. doi: 10.1016/j.jep.2025.119357 39800243

[23] Yang R Liu W Zhou Y Cheng B Liu S Wu R . Modulating HIF-1α/HIF-2α homeostasis with Shen-Qi-Huo-Xue formula alleviates tubular ferroptosis and epithelial-mesenchymal transition in diabetic kidney disease. J Ethnopharmacol. (2025) 343:119478. doi: 10.1016/j.jep.2025.119478 39947365

[24] Chen P Zhu ZZ Lang JM Wei A Chen F . Clinical observation on effect of Yiqi Yangyin Huoxue Tongfu principle in treating diabetes mellitus type 2 of secondary failure to sulfonylurea agents. Zhongguo Zhong Xi Yi Jie He Za Zhi. (2004) 24(7):585–8. 15307693

[25] Wang B Liu X Wang T . Study of prevention and treatment on acute radioactive.

[26] Zhou M Ye C Liang Q Pei Q Xu F Wen H . Yiqi Yangyin Huoxue Method in Treating IdiopathicPulmonary Fibrosis: A Systematic Review and Meta-Analysis of Randomized Controlled Trials. Evid Based Complement Alternat Med. (2020) 2020:8391854. doi: 10.1155/2020/8391854 33062025 PMC7548958

[27] Zhang K Wang Z Zhang L Wu H Liu J Zhang M . Pharmacologically inherited carbon dots from Salvia miltiorrhiza with potent antioxidant activity and multi-pathway modulation for myocardial ischemia-reperfusion injury therapy. Theranostics. (2026) 16(4):1855–76. doi: 10.7150/thno.123141 41356205 PMC12680534

[28] Ye L Huang J Liang X Guo W Sun X Shao C . Jiawei Taohe Chengqi Decoction attenuates CCl(4) induced hepatic fibrosis by inhibiting HSCs activation via TGF-β1/CUGBP1 and IFN-γ/Smad7 pathway. Phytomedicine. (2024) 133:155916. doi: 10.1016/j.phymed.2024.155916 39094440

[29] Huang Y Wang ZL He Y Ye LM Guo WQ Zhang JJ . Jiawei Taohe Chengqi Decoction attenuates hepatic fibrosis by preventing activation of HSCs through regulating Src/ERK/Smad3 signal pathway. J Ethnopharmacol. (2023) 305:116059. doi: 10.1016/j.jep.2022.116059 36549368

[30] Shao C Xu H Sun X Pan Y Liang X Huang J . Jiawei Taohe Chengqi decoction inhibition of the notch signal pathway affects macrophage reprogramming to inhibit HSCs activation for the treatment of hepatic fibrosis. J Ethnopharmacol. (2024) 321:117486. doi: 10.1016/j.jep.2023.117486 38030027

[31] Jia Q Zhang X Hao G Zhao Y Lowe S Han L . Tongluo Yishen Decoction Ameliorates Renal Fibrosis via NLRP3-Mediated Pyroptosis *In Vivo* and *In Vitro*. Front Pharmacol. (2022) 13:936853. doi: 10.3389/fphar.2022.936853 35873572 PMC9298980

[32] Wu QH Li PC Zhang FH Hua Z Zhang XH Sun ZX . To evaluate the efficacy of Yishen Tongluo decoction combined with low-dose tadalafil in the treatment of diabetic erectile dysfunction with kidney deficiency and blood stasis syndrome. Zhonghua Nan Ke Xue. (2023) 29(6):527–32. 38602726

[33] Liu Z Shang Q Li H Fang D Li Z Huang Y . Exploring the possible mechanism(s) underlying the nephroprotective effect of Zhenwu Decoction in diabetic kidney disease: An integrated analysis. Phytomedicine. (2023) 119:154988. doi: 10.1016/j.phymed.2023.154988 37523837

[34] Mok HL Cheng KW Xu Y Huang C Lyu C Xu J . Modified Zhenwu Decoction suppresses chronic colitis via targeting macrophage CCR2/Fyn/p38 MAPK signaling axis. Phytomedicine. (2024) 129:155694. doi: 10.1016/j.phymed.2024.155694 38733904

[35] Liu X Liu L Chen P Zhou L Zhang Y Wu Y . Clinical trials of traditional Chinese medicine in the treatment of diabetic nephropathy–a systematic review based on a subgroup analysis. J Ethnopharmacol. (2014) 151(2):810–9. doi: 10.1016/j.jep.2013.11.028 24296085

[36] Zhang L Miao R Yu T Wei R Tian F Huang Y . Comparative effectiveness of traditional Chinese medicine and angiotensin converting enzyme inhibitors, angiotensin receptor blockers, and sodium glucose cotransporter inhibitors in patients with diabetic kidney disease: A systematic review and network meta-analysis. Pharmacol Res. (2022) 177:106111. doi: 10.1016/j.phrs.2022.106111 35183713

