# Supplementary material for: Efficacy and safety of traditional Chinese medicine decoctions in breast cancer treatment: a network meta-analysis
Source: Front Oncol. 2026 Jul 8;16:1785268. doi: 10.3389/fonc.2026.1785268 (PMC13388213; doi:10.3389/fonc.2026.1785268)
Supplement: Supplementary file 1 [file DataSheet1.docx]

Table S1 specific search strategy

((breast neoplasms[MeSH Terms]) OR ((((((((((((((((((((((((((((((((((((((Breast Neoplasms[Title/Abstract]) OR (Breast Neoplasm[Title/Abstract])) OR (Neoplasm, Breast[Title/Abstract])) OR (Neoplasms, Breast[Title/Abstract])) OR (Breast Tumors[Title/Abstract])) OR (Breast Tumor[Title/Abstract])) OR (Tumor, Breast[Title/Abstract])) OR (Tumors, Breast[Title/Abstract])) OR (Breast Cancer[Title/Abstract])) OR (Cancer, Breast[Title/Abstract])) OR (Cancer of Breast[Title/Abstract])) OR (Cancer of the Breast[Title/Abstract])) OR (Malignant Neoplasm of Breast[Title/Abstract])) OR (Breast Malignant Neoplasm[Title/Abstract])) OR (Breast Malignant Neoplasms[Title/Abstract])) OR (Malignant Tumor of Breast[Title/Abstract])) OR (Breast Malignant Tumor[Title/Abstract])) OR (Breast Malignant Tumors[Title/Abstract])) OR (Mammary Cancer[Title/Abstract])) OR (Cancer, Mammary[Title/Abstract])) OR (Cancers, Mammary[Title/Abstract])) OR (Mammary Cancers[Title/Abstract])) OR (Mammary Neoplasms, Human[Title/Abstract])) OR (Human Mammary Neoplasm[Title/Abstract])) OR (Human Mammary Neoplasms[Title/Abstract])) OR (Neoplasm, Human Mammary[Title/Abstract])) OR (Neoplasms, Human Mammary[Title/Abstract])) OR (Mammary Neoplasm, Human[Title/Abstract])) OR (Breast Carcinoma[Title/Abstract])) OR (Breast Carcinomas[Title/Abstract])) OR (Carcinoma, Breast[Title/Abstract])) OR (Carcinomas, Breast[Title/Abstract])) OR (Mammary Carcinoma, Human[Title/Abstract])) OR (Carcinoma, Human Mammary[Title/Abstract])) OR (Carcinomas, Human Mammary[Title/Abstract])) OR (Human Mammary Carcinomas[Title/Abstract])) OR (Mammary Carcinomas, Human[Title/Abstract])) OR (Human Mammary Carcinoma[Title/Abstract]))) AND ((Decoction[Title/Abstract]) OR (Tang[Title/Abstract]))

Table S2 Basic characteristics of the included studies

| Author | Year | Sample size | Mean age | Intervention | Outcomes |
| --- | --- | --- | --- | --- | --- |
| ZF Huang | 2003 | BZT:37  Control:29 | BZT:49  Control:48.5 | BZT:Twice/day，30days | F1;F5 |
| ZL Li | 2025 | BZT:40  Control:40 | BZT:55.28  Control:55.12 | BZT:Twice/day，63days | F1;F2;F3;F5 |
| YC Liu | 2022 | BZT:36  Control:35 | BZT:58.49  Control:58.43 | BZT:Twice/day，2 weeks | F4;F5 |
| FL Wang | 2018 | BZT:64  Control:64 | BZT:53.5  Control:51.3 | BZT:Twice/day，8 AC-T cycles | F3 |
| XB Wang | 2018 | BSHXT:26  Control:26 | BSHXT: 49.56  Control:50.13 | BSHXT: Twice/day，41 days | F3;F5 |
| ZH Zhang | 2019 | BSHXT:41  Control:41 | BSHXT: 49.67  Control:50.61 | BSHXT: Twice/day，16 weeks | F1;F2;F3;F5 |
| J Zhao | 2022 | BSHXT:40  Control:40 | BSHXT: 50.28  Control:50.15 | BSHXT: Twice/day，16 weeks | F5 |
| XY Du | 2023 | CHJLGMLT :59  Control:59 | CHJLGMLT :45.02  Control:45.07 | CHJLGMLT :Twice/day，14 days | F5 |
| Y Yang | 2018 | CHJLGMLT :46  Control:46 | CHJLGMLT :49.12  Control:48.86 | CHJLGMLT :Twice/day，7 days | F5 |
| YZ Chen | 2024 | FJHQT: 50  Control:50 | FJHQT: 43.78  Control:44.12 | FJHQT: Twice/day，84 days | F1;F2;F3;F5 |
| GX Huang | 2018 | FJHQT: 40  Control:40 | FJHQT: 51.7  Control:51.6 | FJHQT: Twice/day，4 weeks | F5 |
| JT Li | 2024 | FJHQT: 52  Control:52 | FJHQT: 46.12  Control:45.25 | FJHQT: Once/day，21days | F5 |
| M Tang | 2018 | FJHQT: 43  Control:43 | FJHQT: 53.7  Control:54.3 | FJHQT: Twice/day，2 weeks | F5 |
| W Wang | 2018 | FJHQT: 30  Control:30 | FJHQT: 41.23  Control:44.62 | FJHQT: Twice/day，14 days | F3 |
| CK Xiong | 2020 | FJHQT: 20  Control:20 | FJHQT: 47.63  Control:49.15 | FJHQT: Twice/day，14 days | F5 |
| Y Yang | 2016 | FJHQT: 47  Control:47 | FJHQT: 53.1  Control:52.2 | FJHQT: Twice/day | F5 |
| HJ Li | 2011 | FZXLT:42  Control:42 | FZXLT:46.1  Control:47.4 | FZXLT: Twice/day，3 month | F3 |
| SF Zhou | 2017 | FZXLT:37  Control:37 | FZXLT:45.86  Control:46.42 | FZXLT: Twice/day，84 days | F3 |
| L Zhang | 2012 | FZXLT:101  Control:99 | FZXLT:48.23  Control:49.27 | FZXLT: Twice/day，90 days | F3 |
| WW Yang | 2024 | FZXLT:25  Control:25 | FZXLT:53.0  Control:51.9 | FZXLT: Twice/day，84 days | F3 |
| L Wang | 2019 | FZXLT:55  Control:52 | FZXLT:56.9  Control:57.8 | FZXLT: Twice/day，18 weeks | F1;F2;;F5 |
| YP Song | 2020 | FZXLT:50  Control:50 | FZXLT:47.18  Control:57.8 | FZXLT: Twice/day，80 days | F3 |
| BT Lv | 2016 | FZXLT:53  Control:53 | FZXLT:52.3  Control:51.8 | FZXLT: Twice/day，84 days | F1;F2 |
| PT Fu | 2023 | FZXLT:50  Control:50 | FZXLT:47.18  Control:47.92 | FZXLT: Twice/day，28 days | F3 |
| JW Gan | 2020 | FZXLT:48  Control:48 | FZXLT:57.5  Control:56.8 | FZXLT: Twice/day，8 weeks | F3 |
| KX Du | 2022 | GPT: :38  Control:38 | GPT: 47.11  Control:47.81 | GPT: Twice/day，12 weeks | F3;F5 |
| X Wan | 2025 | GPT: :35  Control:34 | GPT: 58.97  Control:60.00 | GPT: Twice/day，8 weeks | F3 |
| M Yuan | 2021 | GPT: :77  Control:77 | GPT: 52.41  Control:52.73 | GPT: Twice/day，8 weeks | F3 |
| YF Dai | 2018 | HQJDT:40  Control:40 | HQJDT:63.5  Control:62.3 | HQJDT:Twice/day，3 month | F1;F2 |
| RQ Wang | 2019 | HQJDT:43  Control:43 | HQJDT:55.12  Control:54.51 | HQJDT:Twice/day，3 month | F1;F2 |
| XQ Wang | 2018 | HQJDT:71  Control:71 | HQJDT:53.31  Control:52.94 | HQJDT:Twice/day，126 days | F1;F2 |
| SY Yang | 2019 | HQJDT:51  Control:51 | HQJDT:52.43  Control:51.70 | HQJDT:Twice/day，126 days | F1;F5 |
| YH Feng | 2023 | RYT: 40  Control:40 | RYT: 45.67  Control:44.65 | RYT:Twice/day，63 days | F1;F2;F3 |
| T Wu | 2018 | RYT: 35  Control:35 | RYT: 45.24  Control:44.30 | RYT:Twice/day，63 days | F1;F2;F3;F5 |
| BH Xu | 2012 | RYT: 26  Control:26 | RYT: 40.9  Control:41.2 | RYT:Twice/day，9 weeks | F3 |
| YX Yuan | 2025 | RYT: 44  Control:44 | RYT: 62.07  Control:62.12 | RYT:Twice/day，84 days | F1;F2:F5 |
| LY Cheng | 2023 | THSWT: 42  Control:42 | THSWT: 46.34  Control:46.59 | THSWT:Twice/day，84 days | F4 |
| HY Yang | 2007 | THSWT: 20  Control:18 | THSWT: 45  Control:43 | THSWT:Twice/day，63 days | F1;F5 |
| BJ Li | 2023 | XSLJZT:49  Control:49 | XSLJZT:49.56  Control:49.31 | XSLJZT:Twice/day，84 days | F3 |
| LL Li | 2020 | XSLJZT:23  Control:22 | XSLJZT:47.51  Control:47.57 | XSLJZT:Twice/day，10 days | F5 |
| D Liu | 2018 | XSLJZT:60  Control:60 | XSLJZT:42.65  Control:41.32 | XSLJZT:Twice/day，84 days | F1;F2;F5 |
| DJ Mou | 2023 | XSLJZT:40  Control:40 | XSLJZT:45.54  Control:45.31 | XSLJZT:Twice/day，42 days | F3 |
| MY Wang | 2023 | XSLJZT:32  Control:32 | XSLJZT:47.0  Control:51.8 | XSLJZT:Twice/day，5 days | F5 |
| JB Li | 2015 | XASQT:49  Control:49 | XASQT:47.2  Control:47.2 | XASQT:Twice/day，60 days | F3 |
| DB Liu | 2020 | XASQT:34  Control:34 | XASQT:47.57  Control:47.86 | XASQT:Twice/day，60 days | F1;F2;F3 |
| HY Liu | 2018 | XASQT:47  Control:47 | XASQT:48.21  Control:47.96 | XASQT:Twice/day，2-4 weeks | F1;F2 |
| H Shi | 2013 | XASQT:30  Control:30 | XASQT:46.0  Control:45.0 | XASQT:Twice/day，60 days | F1;F2;F3 |
| YH Wang | 2016 | XASQT:55  Control:55 | XASQT:47.58  Control:48.17 | XASQT:Twice/day，6 days | F1;F3 |
| S Yu | 2017 | XASQT:52  Control:54 | XASQT:50.3  Control:50.4 | XASQT:Twice/day，6 weeks | F1;F2;F3 |
| QX Zhang | 2021 | XASQT:55  Control:55 | XASQT:50.43  Control:50.45 | XASQT:Twice/day，8 weeks | F3 |
| SH Zhu | 2018 | XASQT:51  Control:51 | XASQT:47.29  Control:46.87 | XASQT:Twice/day，60 days | F1;F2;F3 |
| ZH Gan | 2021 | XCHT:30  Control:30 | XCHT:45.26  Control:46.17 | XCHT:Twice/day，28 weeks | F5 |
| XL Huang | 2018 | XCHT:48  Control:48 | XCHT:57.5  Control:59 | XCHT:Twice/day，3 month | F3 |
| HY Lai | 2023 | XCHT:30  Control:30 | XCHT:54.95  Control:54.88 | XCHT:Once/day，6 month | F1;F2 |
| BY Liu | 2019 | XCHT:66  Control:66 | XCHT:42.16  Control:42.19 | XCHT:Twice/day，21 days | F1 |
| YC Luo | 2016 | XCHT:48  Control:48 | XCHT:42.09  Control:41.35 | XCHT:Twice/day，12 month | F5 |
| CH Qin | 2015 | XCHT:22  Control:22 | XCHT:41.2  Control:41.2 | XCHT:Twice/day，3 month | F1;F2;F5 |
| CX Wu | 2023 | XCHT:41  Control:41 | XCHT:52.27  Control:51.43 | XCHT:Twice/day，3 month | F1;F2;F5 |
| SK Chen | 2025 | YHHYT: 31  Control:31 | YHHYT: 40.23  Control:40.13 | YHHYT: Twice/day，2 month | F1;F2;F3 |
| C Zhang | 2018 | YHHYT: 52  Control:52 | YHHYT: 52.94  Control:53.45 | YHHYT: Twice/day，12 weeks | F3;F5 |
| L Feng | 2022 | YHT:30  Control:30 | YHT: 56.8  Control:55.3 | YHT:Twice/day，16 weeks | F1 |
| MH Hu | 2023 | YHT:31  Control:31 | NR | YHT:Twice/day，NAC cycle | F1;F2;F5 |
| Q Huang | 2019 | YHT:30  Control:29 | YHT: 44.3  Control:44.6 | YHT:3 times a day，84 days | F5 |
| Q Huang | 2019 | YHT:30  Control:30 | YHT: 44.3  Control:44.6 | YHT:3 times a day，84 days | F3;F5 |
| Y Li | 2020 | YHT:21  Control:21 | YHT: 59.28  Control:60.44 | YHT:Twice/day，6 month | F1;F2;F5 |
| D Mao | 2023 | YHT:35  Control:35 | YHT: 43.68  Control:42.93 | YHT:Twice/day，12 weeks | F1;F2;F3;F5 |
| MJ Wang | 2017 | YHT:30  Control:30 | YHT: 48.51  Control:48.11 | YHT:Twice/day，42 days | F1;F2 |
| YF Wu | 2018 | YHT:20  Control:20 | YHT: 60.6  Control:62.2 | YHT:Once/day，21 days | F1;F2 |

BZT: Bazhen Decoction; BSHXT: Bushen Huoxue Decoction; CHJLGMLT : Chaihu Jialonggu Muli Decoction; FJHQT: Fangji Huangqi Decoction; FZXLT: Fuzheng Xiaoliu Decoction; GPT: Guipi Decoction; HQJDT: Huangqi Jiedu Decoction; RYT: Ruyan Decoction; THSWT: Taohong Siwu Decoction; XSLJZT: Xiangsha Liujunzi decoction ; XASQT: Xiaoaishunqi Decoction; XCHT: Xiaochaihu Decoction; YHHYT: Yanghe Huayan Decoction; YHT: Yanghe Decoction; NR: not reported; F1: Objective response rate; F2: Disease control rate; F3: CD4/CD8; F4: C-reactive protein; F5: Nausea and vomiting

1. Chen Shikai, Wang Zudi, and Zhong Zhiguang, Effects of Yanghe Huayan Decoction on Reproductive Hormones and Immune Function in Breast Cancer Patients Resistant to Endocrine Therapy. Chinese Prescription Drugs, 2025. 23(17): pp. 73-76.

2. Chen Yunzi, Yu Haitao, and Chen Yitian, Observation on the Efficacy of Fangjihuangqi Decoction Combined with EC Regimen in Treating Breast Cancer and Its Effects on Serum Lactate Dehydrogenase and Ki-67 Levels. New Chinese Medicine, 2024. 56(14): pp. 158-162.

3. Cheng Lanyun, et al., Effects of Taohong Siwu Decoction on Bcl-2, Bax and Ki-67 Protein Expression in Invasive Breast Cancer with Qi-Stagnation and Blood-Stasis Pattern. Primary Healthcare Chinese Medicine, 2023. 2(4): pp. 34-37.

4. Dai Yongfu, et al., Long-term efficacy analysis of Astragalus detoxification decoction in patients with triple-negative breast cancer. Chinese Journal of Cancer Prevention and Treatment, 2018. 25(S1): pp. 56–57.

5. Du Kexin, Clinical observation of Guipi decoction in treating qi and blood deficiency syndrome after breast cancer surgery. Modern Distance Education in Chinese Medicine, 2022. 20(24): pp. 85–87.

6. Du Xiuying, et al., Efficacy of Modified Chaihu Jia Longgu Muli Decoction in Treating Sleep Disorders Induced by Postoperative Radiochemotherapy in Breast Cancer Patients. China Medical Guide, 2023. 21(29): pp. 145–147.

7. Feng Lei, et al., Yanghe Decoction Combined with Zoledronic Acid in Treating 30 Cases of Yang Deficiency and Cold Aggregation Pattern Breast Cancer Bone Metastasis. Hunan Journal of Traditional Chinese Medicine, 2022. 38(10): pp. 12-16.

8. Feng Yanhong and Zhou Lizhi, Therapeutic Observation of Ruyan Decoction Combined with TX Chemotherapy Regimen in Advanced Triple-Negative Breast Cancer Patients. Journal of Integrative Medicine, 2023. 18(5): pp. 1023–1026, 1050.

9. Fu Peiting, Ma Liyan, and Zhang Yufeng, Effects of Fuzheng Xiaoliu Decoction on Immune Function and Long-term Efficacy in Postoperative Breast Cancer Patients. New Chinese Medicine, 2023. 55(11): pp. 164-168.

10. Gan Jiewen, et al., Clinical Efficacy of Fuzheng Xiaoliu Decoction in Treating Cancer-Related Fatigue Induced by Radiotherapy and Chemotherapy in Breast Cancer Patients. Shenzhen Journal of Integrated Traditional Chinese and Western Medicine, 2020. 30(22): pp. 1-5.

11. Gan Zhuohui, Efficacy observation of Minor Bupleurum Decoction in treating 60 cases of invasive lobular carcinoma of the breast. Journal of Mathematical Medicine, 2021. 34(6): pp. 899-900.

12. Hu Minhao, et al., Clinical efficacy and effects on inflammatory factors of Yanghe Decoction in patients undergoing neoadjuvant chemotherapy for breast cancer. Journal of Shanghai University of Traditional Chinese Medicine, 2023. 37(4): pp. 1-8.

13. Huang Guoxian, Clinical Observation on the Treatment of Postoperative Limb Oedema in Breast Cancer Patients Using Modified Fangjihuangqi Decoction. Chinese Journal of Modern Drug Application, 2018. 12(22): pp. 204-206.

14. Huang Qian, et al., The Effect of Yanghe Decoction on Quality of Life in Chemotherapy Patients with Triple-Negative Breast Cancer. Clinical Research in Traditional Chinese Medicine, 2019. 11(15): pp. 80-81.

15. Huang Qian, et al., The Effect of Yanghe Decoction on Immunological Function Indicators in Chemotherapy Patients with Breast Cancer. Clinical Research in Traditional Chinese Medicine, 2019. 11(06): pp. 88-90.

16. Huang Xiaolong, Wang Wei, and Mu Haiou, Effects of Xiaochaihu Decoction Combined with Chemotherapy on Immune Function and Serum Tumour Markers in Advanced Breast Cancer Patients. Chinese Journal of Medical Laboratory Science, 2018. 28(21): pp. 2619-2621, 2625.

17. Huang Zhifen, et al., A Study on the Treatment of Mid-to-Late Stage Breast Cancer Using Modified Bazhen Decoction Combined with Chemotherapy. Journal of Modern Chinese-Western Medicine Integration, 2003. 12(11): pp. 1123–1124, 1126.

18. Lai Hongyu, Analysis of the Efficacy of Minor Bupleurum Decoction Combined with Intravenous Chemotherapy in Patients with Advanced Triple-Negative Breast Cancer. Modern Diagnosis and Treatment, 2023. 34(19): pp. 2866–2869.

19. Li Baojian, Effects of Xiang Sha Liu Jun Zi Tang on Nutritional Status, Immune Function, and Quality of Life in Breast Cancer Patients Undergoing Postoperative Chemotherapy. Chinese Folk Medicine, 2023. 31(12): pp. 85-88.

20. Li Hongjian, et al., Effects of the Method of Strengthening the Constitution and Eliminating Tumours on Cellular Immune Function in Postoperative Breast Cancer Patients. Journal of Traditional Chinese Medicine, 2011. 17(7): pp. 19-21.

21. Li Jitao, et al., Observation on the Efficacy of Modified Fangjihuangqi Decoction Applied Topically Combined with Warm Acupuncture in Treating Upper Limb Lymphoedema Following Breast Cancer Surgery. Journal of Hubei University of Chinese Medicine, 2024. 26(5): pp. 78-81.

22. Li Jiebao. Effects of Xiaoaishunqi Decoction Combined with CAF Chemotherapy on T-Cell Subpopulations and Vascular Endothelial Growth Factor in 147 Breast Cancer Patients: An Efficacy Analysis. Journal of Hainan Medical College, 2015. 21(3): pp. 381-383.

23. Li Lingling and Zhu Jianjun, Investigation into the Effects of Xiang Sha Liu Jun Zi Tang with Modifications as Adjuvant Therapy in Alleviating Chemotherapy-Related Toxic Side Effects in Breast Cancer Patients. Electronic Journal of Clinical Medicine Literature, 2020. 7(89): pp. 140–141.

24. Li Yang and Huang Lizhong, Effects of Yanghe Decoction on CXCL12/CXCR4 and Downstream Vascular Endothelial Growth Factor in Patients with Kidney Yang Deficiency and Bone Metastasis from Breast Cancer. Oncology Pharmacy, 2020. 10(1): pp. 73–76.

25. Li Zelong and Lu Kai, Observation on the Effect of Bazhen Decoction Combined with Targeted Therapy for Qi and Blood Deficiency Syndrome in HER-2 Positive Breast Cancer. Practical Journal of Integrated Traditional Chinese and Western Medicine, 2025. 25(12): pp. 28-30, 34.

26. Liu Baoyi, Short-Term Efficacy Observation of Xiaochaihu Decoction Adjuvant Chemotherapy for Advanced Breast Cancer. Chinese Medicine Guide, 2019. 17(2): p. 148.

27. Liu Dan, Xie Fengfeng, and Chen Ying, Observation on the Toxicity Reduction and Efficacy Enhancement Effects of Xiang Sha Liu Jun Zi Tang in Breast Cancer Patients Undergoing Chemotherapy. Journal of Hunan University of Chinese Medicine, 2018. 38(4): p. 455-458.

28. Liu Dongbo and Wang Xun, Treatment of 34 Cases of Liver Qi Stagnation Pattern Breast Cancer with Xiao Ai Shun Qi Tang Combined with Chemotherapy. Henan Journal of Traditional Chinese Medicine, 2020. 40(1): pp. 82-85.

29. Liu Haiyong, Analysis of the Effect of Xiao Ai Shun Qi Tang Combined with Radiotherapy on Patients with Liver Qi Stagnation Pattern Early Breast Cancer After Breast-Conserving Surgery. Henan Medical Research, 2018. 27(8): pp. 1407-1409.

30. Liu Yichao, Application of Modified Bazhen Decoction Combined with Conventional Western Medication in Patients After Radical Mastectomy for Breast Cancer. Chinese Journal of Folk Medicine, 2022. 34(19): pp. 94-97.

31. Lü Bingtiao and Dou Yifei, ‘Preoperative Chemotherapy with Fu Zheng Xiao Liu Formula Decoction Combined with CEF Regimen for Advanced Breast Cancer.’ Jilin Journal of Traditional Chinese Medicine, 2016. 36(9): pp. 915-918.

32. Luo Yichang, ‘Effects of Minor Bupleurum Decoction Adjuvant Chemotherapy on Tumour Markers and Angiogenesis Indicators in Advanced Breast Cancer Patients.’ Journal of Practical Traditional Chinese Medicine, 2016. 32(4): pp. 352-353.

33. Mao Dan, Li Ling, and Feng Lei, Efficacy Study of Yanghe Decoction Combined with GT Chemotherapy Regimen for Stage IV Triple-Negative Breast Cancer and Its Effects on Th17/Treg Cell Levels. Jiangxi Journal of Traditional Chinese Medicine, 2023. 54(5): pp. 41-43.

34. Mu Dejun, Investigation into the Regulatory Effects of Modified Xiang Sha Liu Jun Zi Tang on Patients Undergoing Postoperative Chemotherapy for Breast Cancer. Chinese Journal of Practical Rural Doctors, 2023. 30(11): pp. 71-74.

35. Qin Chunhua and Li Fengxia, Short-term efficacy observation of Xiaochaihu Decoction as an adjunct to chemotherapy for advanced breast cancer and its effect on tumour markers. Chinese Journal of Hospital Pharmacy, 2015. 35(15): pp. 1420-1421, 1438.

36. Shi Hua and Zhang Zhisheng, Effects of Xiaoaishunqi Decoction Combined with CAF Chemotherapy on Vascular Endothelial Growth Factor and T-Cell Subpopulations in Patients with Liver-Qi Stagnation Pattern Breast Cancer. Hebei Journal of Traditional Chinese Medicine, 2013. 35(4): pp. 504-506, 525.

37. Song Yuepeng, Syndrome Improvement and Mechanism Analysis of Fuzhengxiaoliu Decoction in Treating Breast Cancer. Sichuan Journal of Traditional Chinese Medicine, 2020. 38(1): pp. 165-168.

38. Tang Min, Clinical value of modified Fangjihuangqi Decoction in treating postoperative limb oedema in breast cancer patients. Inner Mongolia Journal of Traditional Chinese Medicine, 2018. 37(8): pp. 3-4.

39. Wan Yan, et al., Effects of Guipi Decoction Combined with Sini Powder on Breast Cancer-Related Depression and Immune Function. Liaoning Journal of Traditional Chinese Medicine, 2025. 52(10): pp. 64-68.

40. Wang Fenglian, Study on the Improvement of Hair Ultrastructure in Breast Cancer Patients Undergoing Chemotherapy by Bazhen Decoction. Practical Drugs and Clinical Medicine, 2018. 21(7): pp. 794–797.

41. Wang Lei, et al., Clinical Efficacy of Fuzheng Xiaoliu Decoction Combined with Trastuzumab in Treating HER-2-Positive Advanced Breast Cancer and Its Effects on Serum Tumour Marker Levels. Sichuan Journal of Traditional Chinese Medicine, 2019. 37(2): pp. 170–173.

42. Wang Maoyun, et al., Clinical Study on Xiang Sha Liu Jun Zi Tang Combined with Chinese Medicinal Foot Bath for Chemotherapy-Induced Nausea and Vomiting After Breast Cancer Surgery. Chinese Journal of Health Care Medicine, 2023. 25(3): pp. 311-313.

43. Wang Mingjun, Clinical Observation of Yang He Tang Combined with Chemotherapy in 30 Cases of Advanced Breast Cancer. Chinese Journal of Ethnic and Folk Medicine, 2017. 26(9): pp. 110-111.

44. Wang Ruiqin and Wu Pengpeng, Effects of Huangqi Jiedu Decoction on Postoperative Recurrence and Metastasis in Patients with Triple-Negative Breast Cancer. Guangming Traditional Chinese Medicine, 2019. 34(24): pp. 3746-3748.

45. Wang Wei, Kong Liuming, and Jiang Mingqiang, Effects of Fangji Huangqi Decoction on Postoperative Oedema and Immunity in Breast Cancer Patients. World Journal of Traditional Chinese Medicine, 2018. 13(5): pp. 1119–1122.

46. Wang Xianbin, et al., Effects of Bu Shen Huo Xue Tang Combined with Chemotherapy on Immune Function and Quality of Life in Patients with Bone Metastases from Triple-Negative Breast Cancer. Journal of Chinese Medicine, 2018. 24(9): pp. 55–57.

47. Wang Xiaoqing, Effects of Huang Qi Jie Du Tang Combined with GT Chemotherapy Regimen on Efficacy and Survival Rates in Patients with Metastatic Triple-Negative Breast Cancer. Journal of Modern Chinese Medicine, 2018. 27(23): pp. 2583–2585.

48. Wang Yuhua, Efficacy and Effects on T-Cell Subpopulations of Xiaocai Shunqi Decoction Combined with CAF Chemotherapy in Patients with Liver-Qi Stagnation Pattern Breast Cancer. Henan Journal of Traditional Chinese Medicine, 2016. 36(2): pp. 283–285.

49. Wu Chenxiu, Clinical Observation of Xiaochaihu Decoction Combined with Chemotherapy for Recurrent Metastatic Advanced Breast Cancer. Modern Distance Education in Chinese Medicine, 2023. 21(17): pp. 145-147.

50. Wu Tao and Abuduwahabu Abula, Effects of Ruyan Decoction Combined with GP Chemotherapy on Immune Function and Vascular Endothelial Function in Advanced Breast Cancer. World Journal of Traditional Chinese Medicine, 2018. 13(4): pp. 846-849.

51. Wu Yifen, et al., Efficacy of Yanghe Decoction with Rhodiola rosea combined with paclitaxel in advanced triple-negative breast cancer patients. Imaging Research and Medical Application, 2018. 2(13): pp. 251-253.

52. Xiong Can-kai, He Chang-liang, and Cui Guo-qing, Clinical efficacy evaluation of modified Fangjihuangqi Decoction for treating postoperative limb oedema in breast cancer patients. World Medical Abstracts (Continuous Electronic Journal), 2020. 20(2): p. 171,173.

53. Xu Bihong, Li Maoqing, and Luo Yuji. Effects of Nüyanxiaotang on Immune Function in Breast Cancer Patients Undergoing Postoperative Chemotherapy. Liaoning Journal of Traditional Chinese Medicine, 2012. 39(10): pp. 1997–1999.

54. Yang Haiyan, Tong Cailing, and Huang Mei, Clinical Study on Peach Kernel and Four Substances Decoction Combined with Neoadjuvant Chemotherapy for Blood Stasis Obstructing the Interior Pattern of Breast Cancer. Journal of Modern Chinese and Western Medicine Integration, 2007. 16(10): pp. 1327–1328.

55. Yang Senyan, et al., Efficacy of Astragalus Detoxification Decoction Combined with GT Chemotherapy Regimen in Treating Metastatic Triple-Negative Breast Cancer and Its Impact on Patients' Th1/Th2 Cytokine Balance. Journal of Oncology Pharmacy, 2019. 9(2): pp. 312–316, 324.

56. Yang Weiwei and Lü Jiwei, Effects of Fuzheng Xiaoliu Decoction on Immune Function and Long-term Efficacy in Postoperative Breast Cancer Patients. Chinese Journal of Modern Drug Application, 2024. 18(23): pp. 134–137.

57. Yang Yang, Investigation into the Effect of Modified Fangjihuangqi Decoction on Postoperative Limb Oedema in Breast Cancer Patients. Chinese Journal of Continuing Medical Education, 2016. 8(27): pp. 175-176.

58. Yang Yang, Application of Chaihu Jia Longgu Muli Decoction in Breast Cancer Patients with Depression. Chinese Journal of Modern Distance Education in Traditional Chinese Medicine, 2018. 16(21): pp. 113-114.

59. Yu Sheng, Observation on the Efficacy of Xiaoaishunqi Decoction Combined with CAF Chemotherapy Regimen for Liver Qi Stagnation Pattern Breast Cancer and Its Effects on T Cell Subpopulations and VEGF. Journal of Traditional Chinese Medicine of Zhejiang, 2017. 52(09): pp. 666-667.

60. Yuan Min, et al., Effects of Modified Guipi Decoction Combined with Traditional Chinese Medicine Heat Compress Therapy on Recovery in Patients with Qi and Blood Deficiency Syndrome Following Breast Cancer Surgery. Chinese Journal of Experimental Formulary, 2021. 27(21): pp. 150–155.

61. Yuan Yingxia, Nie Yuqian, and Mu Huaiwei, The Impact of Ruyan Decoction Combined with Neoadjuvant Chemotherapy on Short-Term Efficacy, Immune Function, and Survival Outcomes in Patients with Triple-Negative Breast Cancer. Chinese Journal of Pharmacoeconomics, 2025. 20(7): pp. 50–53.

62. Zhang Cheng, Yanghe Huayan Decoction Combined with Chemotherapy for Breast Cancer: Efficacy and Effects on Cellular Immunity and Serum Tumour Markers. Sichuan Journal of Traditional Chinese Medicine, 2018. 36(5): pp. 161-163.

63. Zhang Li, et al., Effects of Fuzheng Xiaoliu Decoction on Cellular Immune Function in Postoperative Breast Cancer Patients. Journal of Modern Chinese-Western Medicine Integration, 2012. 21(3): pp. 229-230.

64. Zhang Qingxu, Li Bing, and Wei Chao, Clinical Observation of Xiao-Ai-Shun-Qi Decoction Combined with Whole-Breast Radiotherapy in Patients with Liver-Qi Stagnation Pattern Early-Stage Breast Cancer Undergoing Breast-Conserving Surgery. Yunnan Journal of Traditional Chinese Medicine and Herbal Medicine, 2021. 42(09): pp. 42-45.

65. Zhang Zhihui, et al., Clinical Efficacy and Effects on Immune Function of Bu Shen Huo Xue Tang Combined with Trastuzumab in the Treatment of Her-2 Positive Advanced Breast Cancer. World Journal of Traditional Chinese Medicine, 2019. 14(4): pp. 993-996.

66. Zhao Jun, Analysis of the Effects of Kidney-Tonifying and Blood-Activating Decoction Combined with Trastuzumab on Patients with Liver-Kidney Yin Deficiency Pattern Her-2 Positive Advanced Breast Cancer. Contemporary Medicine, 2022. 28(6): pp. 16-18.

67. Zhou Shifan, Effects of Fuzheng Xiaoliu Decoction as Adjuvant to Chemotherapy on Postoperative Tumour Marker Levels and Immune Function in Breast Cancer Patients. World Journal of Traditional Chinese Medicine, 2017. 12(7): pp. 1544-1546, 1550.

68. Zhu Shihang and Hu Yanhui, Study on the Efficacy of Xia'ai Shunqi Decoction Combined with Whole-Breast Radiotherapy in 102 Patients with Early-Stage Breast Cancer of Liver Qi Stagnation Pattern Undergoing Breast-Conserving Surgery. Journal of Taishan Medical College, 2018. 39(6): pp. 679-680.

Table S3 Composition of decoction

| Author | Year | Intervention |
| --- | --- | --- |
| ZF Huang | 2003 | BZT: Radix rehmanniae 10g, Radix Angelicae Sinensis 10g, Rhizoma Atractylodis 10g, Poria 12g, Rhizoma Chuanxiong 9g, Radix Paeoniae Alba 12g, Radix Codonopsis 15g, Radix Glycyrrhizae 6G, Radix Astragali 30g, Fructus Ligustri Lucidi 18G, Pericarpium Citri Reticulatae 6g |
| ZL Li | 2025 | BZT: Ligusticum chuanxiong, Angelica sinensis, ginseng, Atractylodes macrocephala, Poria cocos, Radix Paeoniae Alba, radix rehmanniae, and roasted licorice 10 g each |
| YC Liu | 2022 | BZT: Angelica 15 g, Codonopsis pilosula, Poria cocos, Radix Rehmanniae 12 g each, Amomum villosum, Ligusticum chuanxiong, angelica dahuricae 9 g each, licorice 3 G |
| C Qin | 2024 | BZT: Ginseng, Atractylodes macrocephala, Poria cocos, Angelica sinensis, Ligusticum chuanxiong, Radix Paeoniae Alba and Radix Rehmanniae were 10 g each |
| FL Wang | 2018 | BZT: Angelica sinensis 12 g, Ligusticum chuanxiong 12 g, Radix Paeoniae Alba 15 g, roasted licorice 6 g, Rehmannia glutinosa 15 g, ginseng 10 g, Atractylodes macrocephala 15 g, Poria cocos 15 g |
| XB Wang | 2018 | BSHXT: Psoralea corylifolia 15 g, Cistanche deserticola 9 g, safflower 4.5 g, Rehmannia glutinosa 15 g, Angelica pubescens 9 g, Cuscuta chinensis 15 g, Lycium barbarum 9 g, Eucommia ulmoides 9 g, Angelica sinensis tail 9 g, Cornus officinalis 9 g, myrrh 9 g |
| ZH Zhang | 2019 | BSHXT: Fructus Ligustri Lucidi 20 g, Radix Rehmanniae 15 g, Fructus Lycii 15 g, Radix Adenophorae 15 g, Radix Ophiopogonis 15 g, Radix Angelicae Sinensis 15 g, Fructus Vaccariae 15 g, Rhizoma Chuanxiong 12 g, Rhizoma pangolin 8 g |
| J Zhao | 2022 | BSHXT: Ligustrum lucidum 20 g, Lycium barbarum, Rehmannia glutinosa, Radix Adenophorae, Angelica sinensis and wangbuliuxing 15 g each, Ligusticum chuanxiong and pickled pangolin 10 g each |
| XY Du | 2023 | CHJLGMLT : 30 g of raw ochre, oyster, raw keel, 20 g of Polygonum cuspidatum, 15 g of raw Rehmannia glutinosa, 15 g of raw peony, 10 g of Scutellaria baicalensis Georgi, Bupleurum chinense, Poria cocos, gardenia, Pinellia ternata and Curcuma, 6 g of Dangshen and rhubarb, 5 g of ginger and Ramulus Cinnamomi |
| Y Yang | 2018 | CHJLGMLT :Radix Bupleuri, Polygala tenuifolia, Codonopsis pilosula, Acorus tatarinowii, Scutellaria baicalensis, radix curcumae, Pinellia ternata, 15g Poria cocos, 30 g Salvia miltiorrhiza, fried sour jujube kernel, Albizzia julibrissin skin, raw keel, raw oyster, 40 g Longchi, nightshade |
| YZ Chen | 2024 | FJHQT: Coix seed 20 g, stir fried Atractylodes macrocephala, Astragalus membranaceus, Eupatorium adenophorum, Poria cocos peel, Rhizoma alismatis 15 g each, rhizoma curcumae, Rhizoma sparganii, Ramulus Mori, Pericarpium Citri Reticulatae 6 g each, Codonopsis pilosula, Fangji, Angelica sinensis 12 g each, Ramulus Cinnamomi, earthworm 10 g each, licorice 5 g |
| GX Huang | 2018 | FJHQT: Mulberry twig, Sparganium, tangerine peel, rhizoma curcumae, bupleurum, fried Atractylodes macrocephala, Eupatorium adenophorum, Astragalus membranaceus, Rhizoma alismatis, Poria cocos peel, coix seed 18 g, earthworm, Cinnamon Twig 8 g, licorice 4 g, dangshen, Fangji, angelica 10 g each |
| JT Li | 2024 | FJHQT: 9g of white mustard and Euryale ferox, 10g of roasted licorice, 12g of Salvia miltiorrhiza, 15g of Fangji, Atractylodes macrocephala and ginger, 18G of Zhuru and Gentiana macrophylla, 24g of Ramulus Cinnamomi, 30g of Astragalus and Paeonia alba |
| M Tang | 2018 | FJHQT: Coix seed and Astragalus membranaceus were 18G each, Fangji, Alisma orientalis, Poria cocos skin, abdominal skin, Eupatorium adenophorum, fried Atractylodes macrocephala, safflower were 12g each, peach kernel, Angelica sinensis, Codonopsis pilosula, mulberry branch, earthworm were 8g each, ginger peel and licorice were 4G each |
| W Wang | 2018 | FJHQT: Coix seed 25 g, Astragalus membranaceus 20 g, Atractylodes macrocephala 15 g, Poria cocos peel 15 g, Alisma orientalis 15 g, Codonopsis pilosula 12 g, angelica 12 g, Fangji 12 g, earthworm 9 g, zedoary rhizome 9g, peach kernel 9 g, Cinnamon Twig 9 g, tangerine peel 9 g, Bupleurum chinense 9 g |
| CK Xiong | 2020 | FJHQT:Liquorice, bupleurum, tangerine peel, Sparganium, zedoary turmeric, mulberry branches, 5 g each,Cinnamon Twig, Angelica sinensis 10 g each, Codonopsis pilosula and Fangji 12 g each, fried Atractylodes macrocephala, Eupatorium adenophorum, Alisma orientalis, Poria cocos peel, Astragalus membranaceus 15 g each, coix seed 20 g |
| Y Yang | 2016 | FJHQT:Coix seed 18 g, Eupatorium adenophorum, Rhizoma alismatis, Poria cocos peel, fried Atractylodes macrocephala, Astragalus membranaceus 12 g each, Fangji, Angelica sinensis, Codonopsis pilosula 10 g each, Cinnamon Twig, earthworm 8 g each, Sparganium, zedoary turmeric, mulberry twig, bupleurum, tangerine peel 5 g each, licorice 4 g |
| HJ Li | 2011 | FZXLT: American ginseng 10 g, Ganoderma 15 g, raw Astragalus 30 g, Polyporus 30 g, Agrimonia 15 g, Lily 30 g, coix seed 30 g, Scutellaria barbata 10 g, Pinellia ternata 10 g, tangerine peel 10 g, Hedyotis diffusa 15 g, Sparganium 15 g, arrowhead mushroom 10 g, Rhizoma Dioscoreae 5 g, Rhizoma Curcumae 15 g, raw licorice 10 g |
| SF Zhou | 2017 | FZXLT: American ginseng and Ganoderma lucidum were 15g each, Astragalus membranaceus, Polyporus umbellatus, Lily and coix seed were 30 g each, agrimony, Hedyotis diffusa, Sparganium and zedoary rhizome were 18 g each, Scutellaria barbata, tangerine peel, Pinellia ternata, Sagittaria mushroom and Glycyrrhiza uralensis were 10 g each |
| L Zhang | 2012 | FZXLT: American ginseng 10 g, Ganoderma 15 g, raw Astragalus 30 g, Polyporus 30 g, Agrimonia 15 g, Lily 30 g, job's tears 30 g, Scutellaria barbata 10 g, Pinellia ternata 10 g, tangerine peel 10 g, Hedyotis diffusa 15 g, Sparganium 15 g, Sagittaria Sagittaria 10 g, Rhizoma Dioscoreae 5 G, Rhizoma Curcumae 15 g, raw licorice 10 g |
| WW Yang | 2024 | FZXLT: Raw Hawthorn 15 g, Salvia miltiorrhiza 15 g, Patrinia villosa 15 g, Pseudostellaria 10 g, frankincense 15 g, plantain 20 g, Polygonum cuspidatum 20 g, myrrh 20 g, Hedyotis diffusa 20 g, musk 12 g, raw rhubarb 12 g, angelica 12 g, Cuscuta 12 g, fried Atractylodes 12 g, tulip 12 g, roasted Astragalus 30 g, bezoar 6 g |
| L Wang | 2019 | FZXLT: Baked Astragalus 30g, Ganoderma lucidum 20g, arrowhead mushroom 10g, Codonopsis pilosula 30g, fried Atractylodes macrocephala 30g, coix seed 30g, Hedyotis diffusa 10g, LIANLI root 10g, Rhizoma Polygonati 20g, zedoary vinegar 10g |
| YP Song | 2020 | FZXLT: American ginseng 10g, Ganoderma lucidum 15g, Astragalus membranaceus 30g, Polyporus umbellatus 30g, Agrimonia pilosa 15g, Lily 30g, job's tears 30g, Scutellaria barbata 10g, Pinellia ternata 10g, tangerine peel 10g, Hedyotis diffusa 15g, Sparganium sparganii 15g, Sagittaria Sagittaria 10g, dioscorea chinensis 5g, Rhizoma Curcumae 15g, raw licorice 10g |
| BT Lv | 2016 | FZXLT: Astragalus membranaceus, Lily and Codonopsis pilosula 30g each, Agrimonia pilosa, Lycium barbarum and Angelica 20 g each, Ganoderma lucidum, zedoary turmeric, tangerine peel and coix seed 15 g each, raw licorice, Atractylodes macrocephala, Poria cocos, Sagittaria Sagittaria and Scutellaria barbata 10 g each |
| PT Fu | 2023 | FZXLT: American ginseng, Pinellia ternata, Scutellaria barbata, orange peel, arrowhead mushroom and licorice were 10 g each, Ganoderma lucidum, agrimony, Sparganium, Hedyotis diffusa were 15 g each, Astragalus membranaceus, Polyporus umbellatus, lily, coix seed were 30 g each, and Dioscorea opposita was 5 g |
| JW Gan | 2020 | FZXLT: Astragalus membranaceus, Radix Pseudostellariae, Hedyotis diffusa, Scutellaria barbata and coix seed were 30 g each, Ligustrum lucidum, Poria cocos, Atractylodes macrocephala, Ganoderma lucidum and Polyporus umbellatus were 20 g each, tangerine peel and Sagittaria glabra were 10 g each |
| KX Du | 2022 | GPT: Atractylodes macrocephala 9 g, angelica 9 g, Poria 9 g, Astragalus 12 g, longan meat 12 g, wild jujube kernel 12 g, Polygala tenuifolia 6 g, ginseng 6 g, Aucklandia 6 g, ginger 6 g, roasted licorice 3 G, jujube 10 g |
| X Wan | 2025 | GPT: Roasted Astragalus 30g, dangshen 15g, Atractylodes macrocephala 12g, Poria 15g, longan meat 15g, bupleurum 9g, citrus aurantium 12g, Paeonia lactiflora 15g, semen ziphi Spinosae 15g, Angelica sinensis 15g, Polygala tenuifolia 12g, Aucklandia 10g, roasted licorice 6g |
| M Yuan | 2021 | GPT: Stir fried Atractylodes macrocephala 15 g with bran, angelica 10 g, Poria 10 g, Astragalus 30 g, roasted Liquorice 10 g, ginseng 10 g, polygonatum 20 g, Rehmannia glutinosa 30 g, Ligusticum chuanxiong 10 g, tangerine peel 10 g |
| YF Dai | 2018 | HQJDT: Roasted Astragalus 30g, Radix Pseudostellariae 15g, Curcuma 12g, stir fried Atractylodes 12g, Cuscuta 12g, Patrinia villosa 15g, Hedyotis diffusa 20g, angelica 12g, Polygonum cuspidatum 20g, raw rhubarb 12g (not later), Salvia miltiorrhiza 15g, plantain 20g, ginseng Panax Notoginseng Powder 4G (mixed), areca 15g, raw Hawthorn 15g |
| RQ Wang | 2019 | HQJDT: Astragalus membranaceus 30 g, Atractylodes macrocephala 12 g, Hedyotis diffusa, Scutellaria barbata, coix seed 30 g each, Ligustrum lucidum, Solanum nigrum, Radix Pseudostellariae, Radix Scrophulariae 15 g each, Poria cocos, Fritillaria thunbergii, Sagittaria mushroom, Ophiopogon japonicus and Glycyrrhiza uralensis 6 g each |
| XQ Wang | 2018 | HQJDT:Astragalus membranaceus 30 g, Scutellaria barbata 30 g, Hedyotis diffusa 30 g, coix seed 30 g, Solanum nigrum 15 g, Ligustrum lucidum 15 g, Radix Scrophulariae 15 g, Radix Pseudostellariae 15 g, Fritillaria thunbergii 10 g, Poria cocos 10 g, Atractylodes macrocephala 10 g, arrowhead mushroom 10 g, Ophiopogon japonicus 10 g, licorice 6 g |
| SY Yang | 2019 | HQJDT:Astragalus membranaceus, Scutellaria barbata, coix seed, Hedyotis diffusa 30 g each, Radix Pseudostellariae, Radix Scrophulariae, Fructus Ligustri Lucidi, and Solanum nigrum 15 g each, Atractylodes Macrocephalae, Poria cocos, Ophiopogon japonicus, Sagittaria mushroom, Fritillaria thunbergii 10 g each, and Glycyrrhiza uralensis 6 g |
| YH Feng | 2023 | RYT: Semen Coicis 30g, Radix Astragali 25g, Codonopsis pilosula 20g, semen cuscutae, Rhizoma Polygonati, Taraxacum mongolicum, Fructus Lycii, Hedyotis diffusa, Fructus Ligustri Lucidi, Poria cocos 15g each, Radix Scrophulariae 10g, Radix Glycyrrhizae 5g |
| T Wu | 2018 | RYT: Coix seed 30 g, Astragalus 25 g, Codonopsis pilosula 20 g, Taraxacum 15 g, Ligustrum lucidum 15 g, Lycium barbarum 15 g, polygonatum 15 g, Hedyotis diffusa 15 g, Cuscuta chinensis 15 g, Poria cocos 15 g, Scrophularia 10 g, licorice 5 g |
| BH Xu | 2012 | RYT: Astragalus membranaceus 30g, Codonopsis pilosula 24g, Polygonatum sibiricum 15g, Ligustrum lucidum 10g, Cuscuta chinensis 12g, Lycium barbarum 10g, Rhizoma Curcumae 15g, Hedyotis diffusa 24g, coix seed 30g, Scrophularia ningpoensis 12g, Poria cocos 15g, raw licorice 10g |
| YX Yuan | 2025 | RYT: Astragalus membranaceus, Codonopsis pilosula, dandelion, coix seed, Hedyotis diffusa 30 g each, Poria cocos and Polygonatum 15 g each, Ligustrum lucidum, Lycium barbarum, Scrophularia, Cuscuta chinensis 10 g each, licorice 6 g |
| LY Cheng | 2023 | THSWT: Prepared rehmannia glutinosa 12 g, peach kernel, white peony, Angelica sinensis 9 g each, Ligusticum chuanxiong, safflower 6 g each |
| HY Yang | 2007 | THSWT: Peach kernel 15g, safflower 6G, Rehmannia glutinosa 15g, Angelica sinensis 10g, peony 10g, Chuanxiong 8g |
| BJ Li | 2023 | XSLJZT：Poria cocos 15g, stir fried Atractylodes macrocephala, roasted licorice and Aucklandia 10g each, sun dried ginseng and Amomum villosum 6G each |
| LL Li | 2020 | XSLJZT：20 g of Astragalus membranaceus, 15g of jujube, chicken inner golden, Pinellia ternata, yam, Poria cocos, Codonopsis pilosula, 10 g of jiaosanxian, roasted licorice, Pinellia ternata, tangerine peel, Atractylodes macrocephala, Amomum villosum |
| D Liu | 2018 | XSLJZT: Perilla frutescens stem, ginger and bamboo Ru, yam, Atractylodes macrocephala, Poria cocos, orange peel (steamed), fried rice sprouts, fried malt, Magnolia officinalis, cooked Codonopsis pilosula, Fructus aurantii (steamed), hemp seed 15 g each, Amomum villosum 10 g (lower back), and Aucklandia 5 g (lower back) |
| DJ Mou | 2023 | XSLJZT: Raw dried ginseng, Amomum villosum 6 g each, stir fried Atractylodes macrocephala, roasted licorice, wood incense 10 g each, Poria cocos 15 g |
| MY Wang | 2023 | XSLJZT: Dangshen (20 g), Poria Cocos (15 g), Atractylodes macrocephala (15 g) and Astragalus membranaceus (15 g), tangerine peel, Aucklandia, Rhizoma Pinelliae (10 g), Amomum villosum (5 g) and roasted licorice (6 g) |
| JB Li | 2015 | XASQT: Fructus aurantii 6G, seaweed, Curcuma, Paris polyphylla, Cyperus rotundus and bergamot 9g each, Fritillaria thunbergii 10g, Angelica sinensis 12g, Atractylodes macrocephala, Bupleurum chinense, Astragalus membranaceus, Trichosanthes, Taraxacum mongolicum, spatholobus sagittatum and Scutellaria barbata 15g each, Hedyotis diffusa 20g |
| DB Liu | 2020 | XASQT: Hedyotis diffusa 20 g, Scutellaria barbata 15 g, bergamot 9 g, Atractylodes macrocephala 15 g, Paris polyphylla 9 g, Trichosanthes 15 g, seaweed 9 g, bupleurum 15 g, Fructus aurantii 6 g, dandelion 15 g, Fritillaria thunbergii 10 g, Caulis Spatholobi 15 g, Curcuma |
| HY Liu | 2018 | XASQT: Radix Bupleuri, Scutellariae barbatae, cornflower, dandelion, Atractylodes macrocephala, Fructus Trichosanthis, Astragalus membranaceus, Rhizoma Cyperi, radix curcumae, seaweed, Paris polyphylla, bergamot, Fructus aurantii 6G, Hedyotis diffusa 20g, Angelica sinensis 12g, Fritillaria thunbergii 10g |
| H Shi | 2013 | XASQT: Bupleurum 15 g, Cyperus 9 g, angelica 12 g, Atractylodes 15 g, Trichosanthes 15 g, Curcuma 9 g, Paris polyphylla 9 g, Hedyotis diffusa 20 g, Scutellaria barbata 15 g, cornflower 15 g, citrus aurantium 6 g, bergamot 9 g, dandelion 15 g, seaweed 9 g, Fritillaria thunbergii 10 g, Astragalus 15 g |
| YH Wang | 2016 | XASQT: Bupleurum 15 g, Atractylodes 15 g, Scutellaria barbata 15 g, cornflower 15 g, Trichosanthes 15 g, dandelion 15 g, Astragalus 15 g, Rhizoma Cyperi 9 g, Paris polyphylla 9 g, Curcuma 9 g, bergamot 9 g, seaweed 9 g, angelica 12 g, Fructus aurantii 6 g, Fritillaria thunbergii 10 g, Hedyotis diffusa 20g |
| S Yu | 2017 | XASQT: Radix Bupleuri, Atractylodes Macrocephalae, Scutellaria barbata, cornflower, Trichosanthes, dandelion and Astragalus membranaceus were 15g each, Angelica sinensis was 12g, Fritillaria thunbergii was 10g, Cyperus rotundus, Paris polyphylla, Curcuma, bergamot and seaweed were 9g each, Fructus aurantii Immaturus was 6G, Hedyotis diffusa was 20g |
| QX Zhang | 2021 | XASQT: Hedyotis diffusa and Bupleurum chinense 30 g each, Atractylodes macrocephala 10 g, Caulis Spatholobi 10 g, Scutellaria barbata 15 g, Astragalus 20 g, dandelion 10 g, Trichosanthes 10 g, angelica 10 g, Fritillaria thunbergii 10 g, bergamot 10 g, seaweed 10 g, Cyperus 10 g, Curcuma 6 g, Fructus aurantii 6 G |
| SH Zhu | 2018 | XASQT: Hedyotis diffusa 20 g, Atractylodes macrocephala, cornflower, Scutellaria barbata, Astragalus membranaceus, Bupleurum chinense, dandelion and Trichosanthes kirilowii 15 g each, Angelica sinensis 12 g, Fritillaria thunbergii 10 g, bergamot, Paris polyphylla, seaweed, Cyperus, Curcuma 9 g each, citrus aurantium 6 g |
| ZH Gan | 2021 | XCHT: Radix Bupleuri 24g, Radix Scutellariae 9g, Pinellia ternata 9g, ginseng 9g, roasted licorice 9g, ginger 9g, jujube 4 |
| XL Huang | 2018 | XCHT: Medicinal Bupleurum 10 g, Alisma orientalis 10 g, Atractylodes macrocephala 10 g, Scutellaria baicalensis 10 g, Codonopsis pilosula 15 g, Poria cocos 20 g, Ramulus Cinnamomi 5 g, jujube 5 |
| HY Lai | 2023 | XCHT: Ramulus Cinnamomi 5g, roasted licorice 6G, peach kernel 6G, Scutellaria baicalensis Georgi, prepared Pinellia ternata, radix paeoniae rubra, cortex moutan 10g, Radix Bupleuri 12g, Codonopsis pilosula 15g, Poria cocos 20g, jujube 5, ginger 3 |
| BY Liu | 2019 | XCHT: Radix Bupleuri 10 g, Rhizoma Pinelliae 10 g, Radix Scutellariae 10 g, Rhizoma alismatis 10 g, Rhizoma Atractylodis Macrocephalae 10 g, Polyporus umbellatus 20 g, Codonopsis pilosula 15 g, Ramulus Cinnamomi 5 g, Poria cocos 20 g, jujube 5, ginger 3 tablets |
| YC Luo | 2016 | XCHT: Medicinal Bupleurum 10g, Scutellaria 10g, Pinellia ternata 10g, Atractylodes macrocephala 10g, Alisma orientalis 10g, Codonopsis pilosula 15g, Polyporus umbellatus 20g, Poria cocos 20g, Ramulus Cinnamomi 5g, ginger 3 pieces, jujube 5 pieces |
| CH Qin | 2015 | XCHT: Radix Bupleuri, Radix Scutellariae, Rhizoma Pinelliae, Rhizoma Atractylodis Macrocephalae and Rhizoma alismatis 10g each, Codonopsis pilosula 15g, Polyporus umbellatus and poria cocos 20g each, Ramulus Cinnamomi 5g, ginger 3 pieces, jujube 5 pieces |
| CX Wu | 2023 | XCHT: Five jujubes, 10 g bupleurum, 3 slices ginger, 10 g Scutellaria, 5 g Ramulus Cinnamomi, 10 g Pinellia ternata, 20 g Poria cocos, 10 g Atractylodes macrocephala, 20 g Polyporus umbellatus, 15 g Codonopsis pilosula, 10 g Alisma orientalis |
| SK Chen | 2025 | YHHYT: Prepared rehmannia glutinosa 30 g, antler cream 9 g, cinnamon 3 G, ephedra 3 G, white mustard 6 g, ginger charcoal 3 G, licorice 3 G |
| C Zhang | 2018 | YHHYT: Shiitake 15g, antler cream 12g, curcuma zedoary 12g, Rehmannia glutinosa 9g, Fritillaria thunbergii 9g, cinnamon 6G, licorice 6G, white mustard 3G |
| L Feng | 2022 | YHT: Prepared rehmannia glutinosa 30g, antler gum 9g, cinnamon 3G, white mustard 6G, ephedra 2G, ginger charcoal 2G, raw licorice 3G |
| MH Hu | 2023 | YHT: Prepared rehmannia 12 g, cinnamon 3 G, roasted ephedra 6 g, white mustard 12 g, antlers 10 g, ginger charcoal 3 G, raw licorice 3 G |
| Q Huang | 2019 | YHT: Prepared rehmannia glutinosa, cinnamon, ephedra, antler gum, white mustard seed, ginger charcoal, raw licorice, Fritillaria thunbergii, orange peel, hawthorn |
| Q Huang | 2019 | YHT: Prepared rehmannia 15 g, guangui 3 G, roasted ephedra 10 g, antler gum 30 g, white mustard 10 g, ginger charcoal 10 g, Fritillaria thunbergii 10 g, Pinellia ternata 10 g, Poria cocos 10 g, raw licorice 6 g |
| Y Li | 2020 | YHT: Cooked rehmannia, pork hanger, raw licorice |
| D mao | 2023 | YHT: Prepared rehmannia 30 g, antler gum 9 g, cinnamon 3 G, white mustard 6 g, ephedra 2 g, ginger charcoal 2 g, raw licorice 3 G |
| MJ Wang | 2017 | YHT: Prepared rehmannia 30g, cinnamon 3G, ephedra 2G, antler gum 9g, white mustard 6G, ginger charcoal 2G, raw licorice 3G |
| YF Wu | 2018 | YHT: NR |

Table S4 risk bias of summary

| **Study** | **Randomization process** | **Deviations from intended interventions** | **Mising outcome data** | **Measurement of the outcome** | **Selection of the reported result** | **Overall Bias** |
| --- | --- | --- | --- | --- | --- | --- |
| ZF Huang2003 | Low | Low | Low | Some concerns | Low | Some concerns |
| ZL Li2025 | Low | Low | Some concerns | Low | Low | Low |
| YC Liu2022 | Low | Some concerns | Low | Some concerns | Low | Some concerns |
| FL Wang2018 | Low | Low | Some concerns | Low | Some concerns | Some concerns |
| XB Wang2018 | Some concerns | Some concerns | Low | Low | Some concerns | Some concerns |
| ZH Zhang2019 | Some concerns | Low | Low | Low | Low | Low |
| J Zhao2022 | Low | Low | Low | Low | Low | Low |
| XY Du2023 | Low | Some concerns | Low | Low | Some concerns | Some concerns |
| Y Yang2018 | Low | Low | Low | Low | Low | Low |
| YZ Chen2024 | Low | Low | Low | Some concerns | Low | Some concerns |
| GX Huang2018 | Low | Low | Low | Low | Low | Low |
| JT Li2024 | Low | Low | Low | Low | Low | Low |
| M Tang2018 | Low | Some concerns | Low | Low | Some concerns | Some concerns |
| W Wang2018 | Low | Low | Some concerns | Low | Low | Some concerns |
| CK Xiong2020 | Low | Some concerns | Low | Low | Some concerns | Low |
| Y Yang2016 | Low | Low | Low | Low | Low | Low |
| HJ Li2011 | Low | Some concerns | Low | Low | Low | Low |
| SF Zhou2017 | Low | Low | Low | Low | Low | Low |
| L Zhang2012 | Low | Low | Low | Low | Low | Low |
| WW Yang2024 | Low | Low | Some concerns | Low | Low | Some concerns |
| L Wang2019 | Low | Low | Low | Some concerns | Low | Some concerns |
| YP Song2020 | Low | Low | Low | Low | Some concerns | Some concerns |
| BT Lv2016 | Low | Some concerns | Low | Low | Low | Some concerns |
| PT Fu2023 | Low | Low | Some concerns | Low | Low | Some concerns |
| JW Gan2020 | Low | Low | Low | Low | Low | Low |
| KX Du2022 | Low | Low | Low | Some concerns | Low | Some concerns |
| X Wan2025 | Low | Low | Low | Low | Low | Low |
| M Yuan2021 | Low | Some concerns | Low | Some concerns | Low | Some concerns |
| YF Dai2018 | Low | Low | Low | Some concerns | Low | Some concerns |
| RQ Wang2019 | Low | Low | Some concerns | Low | Low | Low |
| XQ Wang2018 | Low | Some concerns | Low | Some concerns | Low | Some concerns |
| SY Yang2019 | Low | Low | Some concerns | Low | Some concerns | Some concerns |
| YH Feng2023 | Some concerns | Some concerns | Low | Low | Some concerns | Some concerns |
| T Wu2018 | Some concerns | Low | Low | Low | Low | Low |
| BH Xu2012 | Low | Low | Low | Low | Low | Low |
| YX Yuan2025 | Low | Some concerns | Low | Low | Some concerns | Some concerns |
| LY Cheng2023 | Low | Low | Low | Low | Low | Low |
| HY Yang2007 | Low | Low | Low | Some concerns | Low | Some concerns |
| BJ Li2023 | Low | Low | Low | Low | Low | Low |
| LL Li2020 | Low | Low | Low | Low | Low | Low |
| D Liu2018 | Low | Some concerns | Low | Low | Some concerns | Some concerns |
| DJ Mou2023 | Low | Low | Some concerns | Low | Low | Some concerns |
| MY Wang2023 | Low | Some concerns | Low | Low | Some concerns | Low |
| JB Li2015 | Low | Low | Low | Low | Low | Low |
| DB Liu2020 | Low | Some concerns | Low | Low | Low | Low |
| HY Liu2018 | Low | Low | Low | Low | Low | Low |
| H Shi2013 | Low | Low | Low | Low | Low | Low |
| YH Wang2016 | Low | Low | Some concerns | Low | Low | Some concerns |
| S Yu2017 | Low | Low | Low | Some concerns | Low | Some concerns |
| QX Zhang2021 | Low | Low | Low | Low | Some concerns | Some concerns |
| SH Zhu2018 | Low | Some concerns | Low | Low | Low | Some concerns |
| ZH Gan2021 | Low | Low | Some concerns | Low | Low | Some concerns |
| XL Huang2018 | Low | Low | Low | Low | Low | Low |
| HY Lai2023 | Low | Low | Low | Some concerns | Low | Some concerns |
| BY Liu2019 | Low | Low | Low | Low | Low | Low |
| YC Luo2016 | Low | Some concerns | Low | Some concerns | Low | Some concerns |
| CH Qin2015 | Low | Low | Low | Low | Low | Low |
| CX Wu2023 | Low | Low | Low | Low | Low | Low |
| SK Chen2025 | Low | Some concerns | Low | Some concerns | Low | Some concerns |
| C Zhang2018 | Low | Low | Low | Low | Low | Low |
| L Feng2022 | Low | Low | Low | Low | Low | Low |
| MH Hu2023 | Low | Some concerns | Low | Some concerns | Low | Some concerns |
| Q Huang2019 | Low | Low | Low | Low | Low | Low |
| Q Huang2019 | Low | Low | Low | Low | Low | Low |
| Y Li2020 | Low | Some concerns | Low | Some concerns | Low | Some concerns |
| D Mao2023 | Low | Low | Low | Low | Low | Low |
| MJ Wang2017 | Low | Low | Low | Low | Low | Low |
| YF Wu2018 | Low | Some concerns | Low | Some concerns | Low | Some concerns |

Table S5 Consistency test results

| Outcomes | Consistency test | Inconsistency test | I^2^(%) |
| --- | --- | --- | --- |
| Objective response rate | 105.06 | 105.21 | 0 |
| Disease control rate | 94.09 | 94.03 | 6 |
| CD4+/CD8+ | 129.75 | 129.81 | 0.7 |
| Nausea and vomiting | 112.77 | 112.78 | 7 |

Table S6 Objective response rate league table

| OR 95%Crl | | | | | | | | | | | | |
| --- | --- | --- | --- | --- | --- | --- | --- | --- | --- | --- | --- | --- |
| BSHXT |  |  |  |  |  |  |  |  |  |  |  |  |
| 0.81(0.56, 1.23) | BZT |  |  |  |  |  |  |  |  |  |  |  |
| 1.83 (0.76, 4.5) | 2.15 (1.06, 4.42) * | Control | 2.27 (1.03, 5.00) * | 2.00 (1.12, 3.57) * | 2.5 (1.64, 3.70) * | 3.13 (1.67, 5.88) * |  | 3.13 (2.00, 4.76) * | 2.44 (1.54, 3.85) * |  | 4.76 (1.25, 25) * | 2.00 (1.23, 3.23) * |
| 0.8 (0.24, 2.67) | 0.94 (0.32, 2.76) | 0.44 (0.2, 0.97) * | FJHQT |  |  |  |  |  |  |  |  |  |
| 0.92 (0.32, 2.67) | 1.08 (0.44, 2.72) | 0.5 (0.28, 0.89) * | 1.15 (0.43, 3.07) | FZXLT |  |  |  |  |  |  |  |  |
| 0.74 (0.28, 1.99) | 0.87 (0.39, 1.99) | 0.4 (0.27, 0.61) * | 0.92 (0.38, 2.27) | 0.8 (0.4, 1.62) | HQJDT |  |  |  |  |  |  |  |
| 0.59 (0.2, 1.75) | 0.69 (0.27, 1.8) | 0.32 (0.17, 0.6) * | 0.73 (0.26, 2.04) | 0.64 (0.27, 1.48) | 0.8 (0.37, 1.67) | RYT |  |  |  |  |  |  |
| 0.35 (0.03, 2.5) | 0.41 (0.04, 2.69) | 0.19 (0.02, 1.08) | 0.44 (0.04, 2.97) | 0.38 (0.04, 2.36) | 0.48 (0.05, 2.8) | 0.6 (0.06, 3.76) | THSWT |  |  |  |  |  |
| 0.59 (0.22, 1.6) | 0.69 (0.3, 1.6) | 0.32 (0.21, 0.5) * | 0.73 (0.29, 1.84) | 0.64 (0.31, 1.31) | 0.8 (0.44, 1.44) | 1 (0.47, 2.16) | 1.67 (0.28, 15.04) | XASQT |  |  |  |  |
| 0.75 (0.28, 2.07) | 0.89 (0.38, 2.08) | 0.41 (0.26, 0.65) * | 0.94 (0.37, 2.4) | 0.82 (0.39, 1.71) | 1.02 (0.55, 1.9) | 1.28 (0.59, 2.82) | 2.14 (0.36, 19.5) | 1.28 (0.68, 2.43) | XCHT |  |  |  |
| 1.06 (0.34, 3.37) | 1.24 (0.45, 3.46) | 0.58 (0.28, 1.2) | 1.32 (0.45, 3.93) | 1.15 (0.45, 2.89) | 1.43 (0.62, 3.29) | 1.8 (0.69, 4.7) | 3.01 (0.46, 28.71) | 1.8 (0.76, 4.2) | 1.4 (0.59, 3.32) | XSLJZT |  |  |
| 0.37 (0.06, 1.92) | 0.44 (0.07, 2.06) | 0.21 (0.04, 0.8) * | 0.46 (0.08, 2.28) | 0.41 (0.07, 1.78) | 0.51 (0.09, 2.09) | 0.64 (0.11, 2.87) | 1.06 (0.1, 13.34) | 0.64 (0.12, 2.65) | 0.5 (0.09, 2.11) | 0.35 (0.06, 1.66) | YHHYT |  |
| 0.92 (0.34, 2.56) | 1.08 (0.46, 2.57) | 0.5 (0.31, 0.81) * | 1.15 (0.45, 2.95) | 1 (0.47, 2.11) | 1.25 (0.66, 2.33) | 1.56 (0.71, 3.46) | 2.6 (0.44, 23.82) | 1.56 (0.81, 3) | 1.22 (0.62, 2.39) | 0.87 (0.36, 2.11) | 2.46 (0.58, 13.65) | YHT |

*Means P<0.05

Table S7 Disease control rate league table

| OR 95%Crl | | | | | | | | | | | |
| --- | --- | --- | --- | --- | --- | --- | --- | --- | --- | --- | --- |
| BSHXT |  |  |  |  |  |  |  |  |  |  |  |
| 0.78 (0.09, 6.23) | BZT |  |  |  |  |  |  |  |  |  |  |
| 4.46 (1.2, 23.59) | 5.77 (1.59, 29)* | Control | 2.78 (1.08, 7.69) |  | 4.00 (2.04, 8.33) | 2.78 (1.16, 6.67) | 4.17 (1.96, 10.00) | 4.35 (2.33, 8.33) | 5.00 (1.14, 33.33 |  | 2.56 (1.28, 5.56) |
| 1.62 (0.3, 10.74) | 2.09 (0.4, 13.43) | 0.36 (0.13, 0.93)* | FJHQT |  |  |  |  |  |  |  |  |
| 5.27 (1.15, 31.93)* | 6.79 (1.52, 39.73)* | 1.17 (0.56, 2.44) | 3.26 (0.98, 11.33) | FZXLT |  |  |  |  |  |  |  |
| 1.11 (0.24, 6.66) | 1.44 (0.32, 8.26) | 0.25 (0.12, 0.49)* | 0.69 (0.21, 2.34) | 0.21 (0.08, 0.58) * | HQJDT |  |  |  |  |  |  |
| 1.63 (0.33, 10.32) | 2.12 (0.43, 12.9) | 0.36 (0.15, 0.86)* | 1.01 (0.27, 3.81) | 0.31 (0.1, 0.96) * | 1.46 (0.47, 4.5) | RYT |  |  |  |  |  |
| 1.08 (0.22, 6.59) | 1.4 (0.3, 8.14) | 0.24 (0.1, 0.51)* | 0.67 (0.19, 2.38) | 0.21 (0.07, 0.59) * | 0.97 (0.33, 2.76) | 0.66 (0.2, 2.16) | XASQT |  |  |  |  |
| 1.03 (0.24, 6.03) | 1.34 (0.31, 7.43) | 0.23 (0.12, 0.43)* | 0.64 (0.2, 2.1) | 0.2 (0.07, 0.51) * | 0.93 (0.36, 2.4) | 0.63 (0.21, 1.91) | 0.95 (0.35, 2.7) | XCHT |  |  |  |
| 0.87 (0.08, 8.1) | 1.13 (0.1, 10.38) | 0.2 (0.03, 0.88) * | 0.54 (0.06, 3.36) | 0.17 (0.02, 0.89) * | 0.79 (0.09, 4.21) | 0.53 (0.06, 3.14) | 0.81 (0.09, 4.53) | 0.85 (0.1, 4.46) | XSLJZT |  |  |
| 1.13 (0.1, 11.51) | 1.46 (0.13, 14.27) | 0.25 (0.03, 1.29) | 0.7 (0.07, 4.87) | 0.21 (0.02, 1.3) | 1.01 (0.11, 6.06) | 0.69 (0.07, 4.54) | 1.05 (0.11, 6.56) | 1.09 (0.12, 6.43) | 1.29 (0.1, 17.45) | YHHYT |  |
| 1.74 (0.38, 10.48) | 2.25 (0.5, 12.87) | 0.39 (0.18, 0.78) * | 1.08 (0.33, 3.7) | 0.33 (0.12, 0.92) * | 1.56 (0.57, 4.31) | 1.07 (0.34, 3.39) | 1.61 (0.56, 4.78) | 1.69 (0.64, 4.42) | 1.98 (0.37, 17.14) | 1.55 (0.25, 14.15) | YHT |

*Means P<0.05

Table S8 CD4+/CD8+ league table

| MD 95%Crl | | | | | | | | | | | |
| --- | --- | --- | --- | --- | --- | --- | --- | --- | --- | --- | --- |
| BSHXT |  |  |  |  |  |  |  |  |  |  |  |
| 0.12 (-0.51, 0.75) | BZT |  |  |  |  |  |  |  |  |  |  |
| 0.44 (0, 0.88) | 0.32 (-0.13, 0.77) | Control | 0.69(0.2, 1.17) |  | 0.42(0.04,0.8) | 0.55(0.18, 0.93) |  |  |  |  |  |
| -0.25 (-0.9, 0.41) | -0.37 (-1.02, 0.29) | -0.69 (-1.17, -0.2)* | FJHQT |  |  |  |  |  |  |  |  |
| 0.19 (-0.32, 0.7) | 0.07 (-0.44, 0.58) | -0.25 (-0.5, 0) | 0.44 (-0.11, 0.98) | FZXLT |  |  |  |  |  |  |  |
| 0.02 (-0.56, 0.6) | -0.1 (-0.69, 0.49) | -0.42 (-0.8, -0.04)* | 0.27 (-0.35, 0.88) | -0.17 (-0.62, 0.28) | GPT |  |  |  |  |  |  |
| -0.11 (-0.7, 0.47) | -0.23 (-0.82, 0.35) | -0.55 (-0.93, -0.18) * | 0.14 (-0.48, 0.75) | -0.3 (-0.75, 0.15) | -0.14(-0.67, 0.4) | RYT |  |  |  |  |  |
| 0.48 (-0.03, 0.98) | 0.36 (-0.16, 0.87) | 0.04 (-0.21, 0.28) | 0.72 (0.18, 1.26) | 0.29 (-0.07, 0.63) | 0.45 (0, 0.91) | 0.59 (0.14, 1.03) * | XASQT |  |  |  |  |
| 0.1 (-0.67, 0.88) | -0.02 (-0.79, 0.75) | -0.34 (-0.97, 0.3) | 0.35 (-0.45, 1.15) | -0.09 (-0.76, 0.6) | 0.08 (-0.66, 0.82) | 0.21 (-0.52, 0.95) | -0.37 (-1.05, 0.31) | XCHT |  |  |  |
| 0.26 (-0.38, 0.89) | 0.14 (-0.5, 0.78) | -0.18 (-0.64, 0.27) | 0.51 (-0.16, 1.17) | 0.07 (-0.45, 0.59) | 0.24 (-0.36, 0.83) | 0.37 (-0.22, 0.96) | -0.22 (-0.73, 0.3) | 0.16 (-0.62, 0.94) | XSLJZT |  |  |
| 0.37 (-0.28, 1.01) | 0.25 (-0.4, 0.9) | -0.07 (-0.54, 0.4) | 0.62 (-0.06, 1.29) | 0.18 (-0.35, 0.71) | 0.35 (-0.26, 0.95) | 0.48 (-0.12, 1.08) | -0.1 (-0.64, 0.43) | 0.27 (-0.52, 1.06) | 0.11 (-0.55, 0.77) | YHHYT |  |
| 0.17 (-0.47, 0.8) | 0.05 (-0.58, 0.68) | -0.27 (-0.72, 0.18) | 0.42 (-0.24, 1.07) | -0.02 (-0.53, 0.49) | 0.15 (-0.44, 0.74) | 0.28 (-0.3, 0.87) | -0.31 (-0.82, 0.21) | 0.07 (-0.71, 0.84) | -0.09 (-0.73, 0.55) | -0.2 (-0.85, 0.45) | YHT |

*Means P<0.0

Table S9 nausea and vomiting league table

| OR 95%Crl | | | | | | | | | | | | |
| --- | --- | --- | --- | --- | --- | --- | --- | --- | --- | --- | --- | --- |
| BSHXT |  |  |  |  |  |  |  |  |  |  |  |  |
| 1.45 (0.5, 4.34) | BZT |  |  |  |  |  |  |  |  |  |  |  |
| 1.54 (0.29, 13.34) | 1.06 (0.18, 9.73) | CHJLGMLT |  |  |  |  |  |  |  |  |  |  |
| 0.36 (0.19, 0.69)* | 0.25 (0.1, 0.57)* | 0.24 (0.03, 1.07) | Control | 0.27 (0.12, 0.57) |  |  |  |  |  | 0.52 (0.28, 0.97) | 0.32 (0.13, 0.76) | 0.30 (0.17, 0.57) |
| 1.34 (0.49, 3.76) | 0.92 (0.29, 2.91) | 0.87 (0.1, 4.89) | 3.69 (1.76, 8.41)* | FJHQT |  |  |  |  |  |  |  |  |
| 0.36 (0.12, 1.02) | 0.25 (0.07, 0.8)* | 0.23 (0.03, 1.32) | 0.99 (0.43, 2.29) | 0.27 (0.08, 0.82)* | FZXLT |  |  |  |  |  |  |  |
| 1.38 (0.24, 12.21) | 0.96 (0.15, 9.04) | 0.89 (0.07, 11.66) | 3.82 (0.76, 31.21) | 1.04 (0.17, 9.54) | 3.9 (0.62, 36.86) | GPT |  |  |  |  |  |  |
| 0.51 (0.09, 3.2) | 0.35 (0.05, 2.37) | 0.32 (0.02, 3.33) | 1.41 (0.28, 7.98) | 0.38 (0.06, 2.5) | 1.43 (0.23, 9.75) | 0.36 (0.03, 3.96) | HQJDT |  |  |  |  |  |
| 0.93 (0.25, 3.73) | 0.64 (0.15, 2.84) | 0.6 (0.06, 4.28) | 2.57 (0.84, 8.86) | 0.69 (0.17, 2.93) | 2.61 (0.65, 11.55) | 0.67 (0.06, 5.17) | 1.83 (0.23, 14.17) | RYT |  |  |  |  |
| 0.51 (0.19, 1.35) | 0.35 (0.11, 1.06) | 0.33 (0.04, 1.8) | 1.41 (0.68, 2.96) | 0.38 (0.13, 1.1) | 1.43 (0.47, 4.37) | 0.37 (0.04, 2.19) | 1 (0.15, 5.93) | 0.55 (0.13, 2.1) | XCHT |  |  |  |
| 0.69 (0.28, 1.71) | 0.48 (0.16, 1.35) | 0.45 (0.05, 2.33) | 1.91 (1.03, 3.62) * | 0.52 (0.18, 1.37) | 1.94 (0.68, 5.54) | 0.5 (0.06, 2.86) | 1.36 (0.22, 7.84) | 0.74 (0.19, 2.67) | 1.36 (0.52, 3.55) | XSLJZT |  |  |
| 1.14 (0.39, 3.45) | 0.79 (0.23, 2.66) | 0.74 (0.08, 4.37) | 3.15 (1.33, 7.8)* | 0.85 (0.26, 2.76) | 3.21 (0.96, 11.01) | 0.82 (0.09, 5.29) | 2.23 (0.32, 14.44) | 1.23 (0.27, 5.18) | 2.24 (0.71, 7.14) | 1.65 (0.56, 4.93) | YHHYT |  |
| 1.2 (0.51, 2.79) | 0.83 (0.29, 2.23) | 0.78 (0.09, 3.91) | 3.31 (1.94, 5.8)* | 0.9 (0.34, 2.26) | 3.37 (1.24, 9.22)* | 0.87 (0.1, 4.79) | 2.36 (0.38, 13.11) | 1.29 (0.34, 4.5) | 2.36 (0.94, 5.89) | 1.73 (0.75, 3.98) | 1.05 (0.37, 2.95) | YHT |

*Means P<0.05


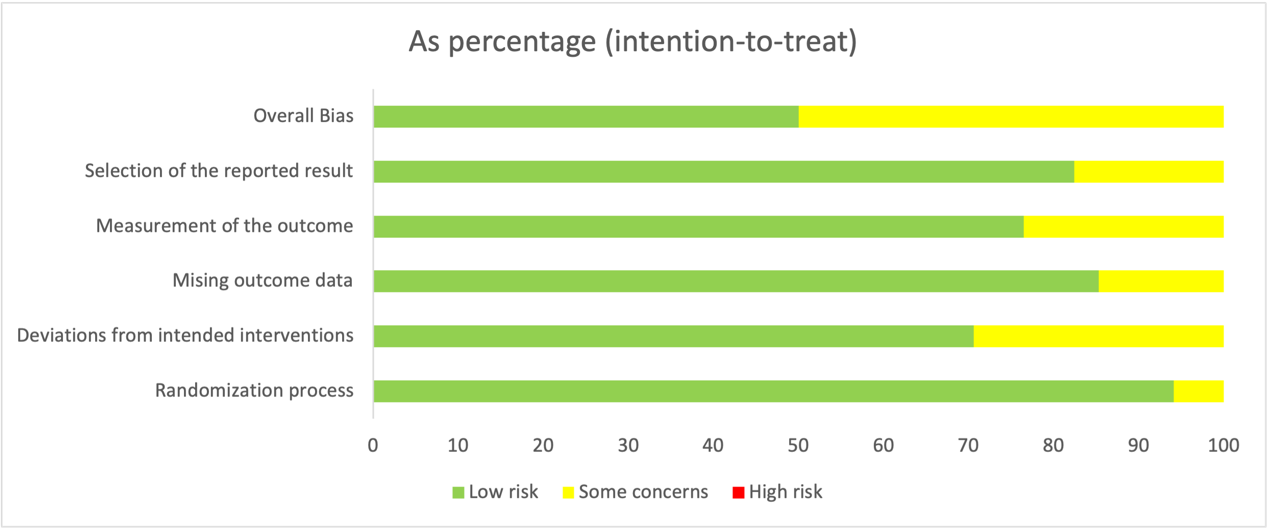


Figure S1 risk of bias graph

Figure S2 Funnel plot of objective response rate

Figure S3 Funnel plot of disease control rate

Figure S4 Funnel plot of CD4+/CD8+

Figure S5 Funnel plot of Nausea and vomiting
